# An integrated gut phage catalogue of nonhuman primates reveals functional adaptation to host ecology and evolutionary link to humans

**DOI:** 10.1098/rspb.2026.0827

**Published:** 2026-11-04

**Authors:** Shengzhi Yang, Lei Jin, Wenwen Deng, Hongtao Xiao, Yongfang Yao, Meng Xie, Huailiang Xu, LiKou Zou

**Affiliations:** ^1^ College of Life Science, Sichuan Agricultural University, Ya'an, Sichuan, People's Republic of China; ^2^ College of Resources, Sichuan Agricultural University, Chengdu, Sichuan, People's Republic of China; ^3^ Key Laboratory of State Forestry and Grassland Administration (SFGA) on Conservation Biology of Rare Animals in the Giant Panda National Park, The China Conservation and Research Center for the Giant Panda (CCRCGP), Chengdu, Sichuan, People's Republic of China

**Keywords:** nonhuman primates, gut phage, gut microbiome, host clade, dietary niche, lifestyle

## Abstract

Nonhuman primates (NHPs), the closest evolutionary relatives of humans, are pivotal models for biomedical and ecological research. Gut phage are recognized as critical modulators of gut microbial communities; however, comprehensive insight into gut phage of NHPs is lacking. We used gut metagenomes spanning 25 NHP species and constructed an integrated gut phage catalogue (IGPC) comprising 11 389 nonredundant phage genomes. Our results revealed that the composition and function of NHP gut phage were significantly influenced by host clade, dietary niche and lifestyle. Host clade exerted a stronger influence than dietary niche on gut phage diversity. The lifestyle of NHPs influenced gut phage diversity and altered auxiliary metabolic gene profiles for different NHP species. Comparatively, 78.5% of IGPC phages were detected in human guts, with higher prevalence in nonindustrialized human guts. crAss-like phage—the most prevalent human-associated phage—were abundant in NHP guts, while exhibiting distinct evolutionary differences from crAss-like phage of humans. These findings comprehensively characterize the gut phageome of NHPs, revealing NHP host-driven and environment-driven functional adaptations, and provide an evolutionary understanding of the gut phageome between NHPs and humans.

## 1. Introduction

Animal guts are colonized by numerous commensal microorganisms, including bacteria, archaea, fungi, viruses and protozoa, collectively termed the gut microbiome, which plays a key role in host health and metabolism [1]. The vast majority of animal gut microbial studies have focused on bacteria because of their greater culturability and well established databases; however, phage (prokaryotic viruses) are increasingly recognized to play a non-negligible role in animal guts. Temperate phage can integrate genetic material into their host genomes in a dormant state and do not cause bacterial lysis [2], while virulent phage can infect and lyse their hosts for reproduction, playing key roles in regulating bacterial communities [3]. Additionally, auxiliary metabolic genes (AMGs) of phage can modulate the metabolism of their hosts, thereby indirectly influencing the composition of bacterial communities [4].

Nonhuman primates (NHPs) are the closest evolutionary relatives of humans and indispensable models in disease research and drug development [5]. Recent studies on NHPs’ gut microbiome have focused primarily on gut bacterial composition and function, revealing that gut bacteria exhibit remarkable plasticity in response to different dietary structures or living environments [6,7]. These studies primarily characterized the gut bacterial features of NHPs, overlooking the important roles of gut phage in these NHP species.

Gut microbial dysbiosis in NHPs is tightly linked to host diseases, such as diarrhoea [8]. While antibiotics remain a primary therapeutic intervention for gut diseases, such as diarrhoea, their overuse may exacerbate the loss of gut microbial diversity and accelerate the emergence of antibiotic resistance [8]. In this context, phage therapy, using the host-specific nature of phage, represents a promising alternative to antibiotic therapy and can precisely regulate gut bacterial communities without disrupting the commensal microbiome and causing antibiotic resistance [9]. Phage-mediated modulation holds immense potential for the treatment of human gastrointestinal diseases [10]. Therefore, understanding gut phage in NHPs can enable the utilization of NHPs as ideal research models for phage therapy in human gut diseases.

With the rapid development of high-throughput sequencing, numerous novel phage genomes have been retrieved from metagenomic datasets covering various environments, such as soil, lake, ocean and animal gut [11]. Although gut phage genomes from NHPs remain scarce, recovering phage genomes from metagenomes can serve as an important approach to significantly advance the in-depth exploration of phage diversity and biological function in NHP guts. Gogarten *et al.* [12] reported that the gut phage compositions of primates were shaped by host phylogeny and environment; furthermore, the captivity of NHPs shifted the gut phageome toward a human-associated state. However, a comprehensive genome-resolved catalogue of gut phages across different NHPs, enabling detailed functional profiling and large-scale evolutionary comparison with humans, is still lacking.

Consequently, we analysed 454 metagenomes from 25 NHP species to characterize gut phage in NHPs and present an integrated gut phage catalogue (IGPC) comprising 11 389 nonredundant phage genomes. Our results suggest that host clade, dietary niche, and lifestyle significantly influence the diversity, composition and function of NHP gut phage. crAss-like phage between NHPs and humans exhibit distinct evolutionary differences. These phage genomic resources provide a comprehensive insight into the gut phageome dynamics of NHPs and contribute to an evolutionary understanding of the gut phageome in primates.

## 2. Material and methods

### (a) Collection of nonhuman primate gut metagenomes

To present a comprehensive overview of the gut phageome in NHPs, we analysed 454 gut metagenomes from 25 NHP species across 13 studies [8,13–24] (figure 1A; supplementary material, table S1). Those samples that were explicitly described as ‘diseased’ or ‘clinically abnormal’ in these studies were excluded. These NHPs were grouped into captive (living in captivity), wild (living independently in wild habitats) and semiwild (living in wild habitats but receiving artificial food provisions) populations (supplementary material, table S2). According to their dietary habits, these NHPs were classified into folivores and nonfolivores. We classified these NHPs into four clades according to genetic relationships: Hominoidea, Cercopithecidae, Platyrrhini and Lemuriformes.

**Figure 1.**
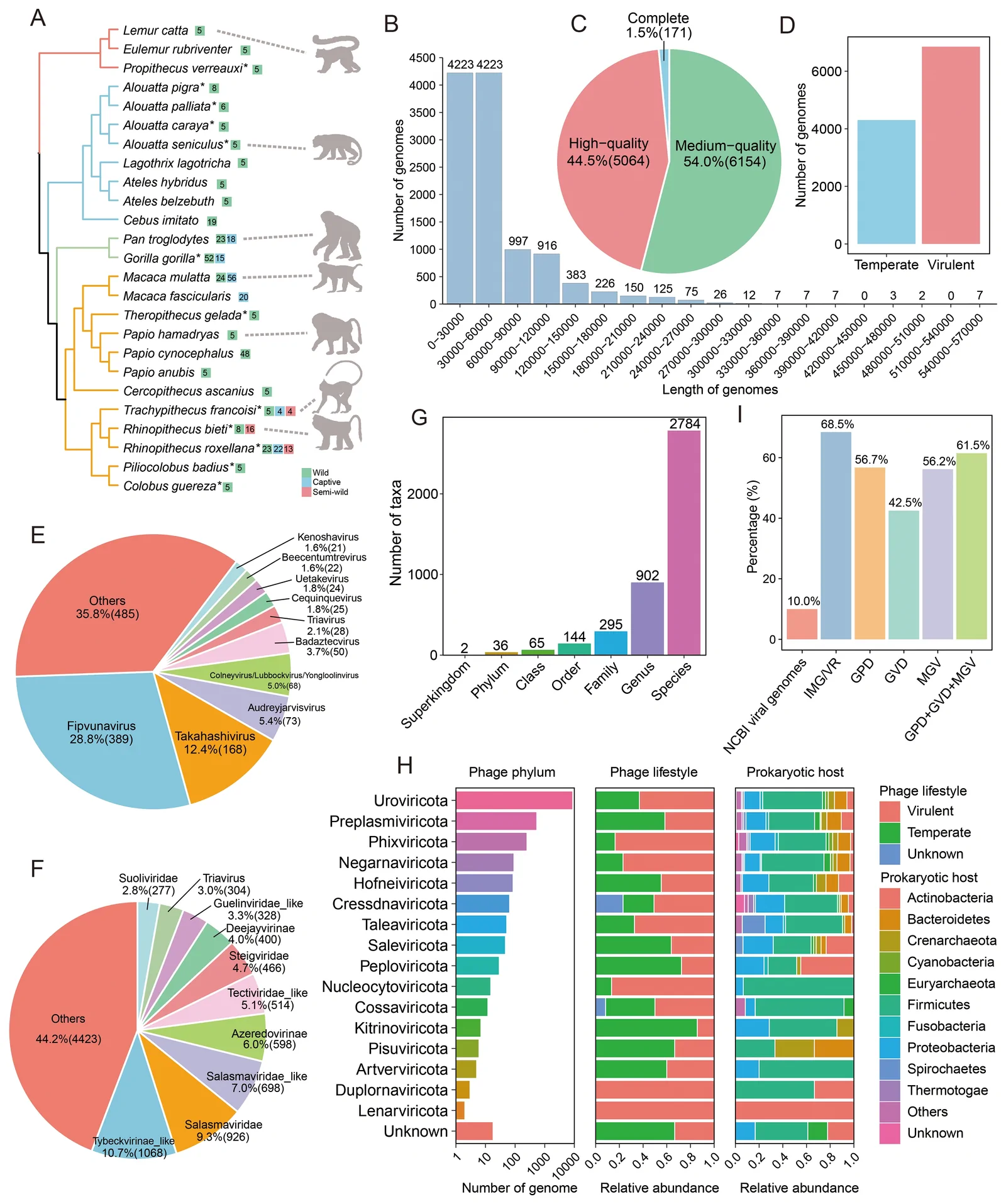
Characterization of phage genomes in the integrated gut phage catalogue (IGPC). (A) Phylogenetic tree of 25 species of nonhuman primates (NHPs) studied. The tree represents only the branching order and does not reflect branch lengths. Species with an asterisk are folivores, and those without are nonfolivores. The number after the species name denotes wild (green), captive (blue) or semiwild (red) sample number. (B) Length and number of phage genomes in IGPC. (C) Percentage and number of complete, high-quality and medium-quality phage genomes in IGPC. (D) Number of temperate phage and virulent phage in IGPC. (E) Percentage and number of phage classified at the genus level (1353 of 11 389 total genomes). (F) Percentage and number of classified families in IGPC phage. (G) Taxonomic numbers of prokaryotic hosts of phage in IGPC at different taxonomic levels. (H) Number, lifestyle and prokaryotic host in different phyla of phage in IGPC. (I) Percentage of IGPC phage with average nucleotide identity >95% compared with NCBI viral reference genomes, IMG/VR v4 database, GVD, GPD and MGV databases.

### (b) Assembly and identification of phage contigs

Adapters and low-quality reads in raw data were removed via Trimmomatic [25]. Host reads were removed via Bowtie 2 [26] using the reference genomes of the hosts or their closely related species from NCBI genome datasets. High-quality metagenomic reads were assembled via MEGAHIT [27] to obtain contigs. The contigs were identified via VirSorter2 [28] with the option ‘–viral-gene-required –min-score 0.7 –min-length 1500’ and the default ‘–include-groups dsDNAphage, ssDNA’. These identified contigs were further verified via DeepVirFinder [29], and the contigs with score >0.9 and *p*-value <0.05 were regarded as putative phage contigs. The quality of the putative phage contigs was estimated via CheckV [30]. All contigs estimated as complete, high-quality and medium-quality were clustered via CD-HIT [31] to obtain a nonredundant gut phage catalogue.

### (c) Annotations of phage lifestyles, prokaryote hosts and taxa

Phage lifestyles were predicted via PhaTYP [32]. Phage hosts were predicted via CHERRY [33]. Phage taxonomic labels at family and genus levels were predicted via PhaGCN2 [34] and PhaGenus [35], respectively.

### (d) Auxiliary metabolic gene annotation of phage genomes

Phage AMGs were functionally annotated via DRAM-v [36] using KOfam and other default databases. The annotated result was filtered via the ‘distill’ function in DRAM-v.

### (e) Comparison of the integrated gut phage catalogue genomes with public viral databases

We compared the IGPC genomes with the NCBI viral reference genomes [37], the IMG/VR v4 database [38] and three human viral databases (Gut Virome Database (GVD) [39], Gut Phage Database (GPD) [40] and Metagenomic Gut Virus (MGV) database [41]). These sequences were locally aligned via BLASTN, and the average nucleotide identity (ANI) between pairwise sequences was calculated via the script ‘anicalc.py’ of CheckV.

### (f) Abundance quantification of phage and bacteria in metagenomes

The abundance of the IGPC phage in NHP gut metagenomes was quantified via CoverM [42] using the ‘coverm contig’ mode and normalized using the transcripts per million (TPM) method. We downloaded 80 gut metagenomes of humans from industrialized (*n* = 39) or nonindustrialized (*n* = 41) countries [43–49]. The abundance of the IGPC phage in humans’ gut metagenomes was quantified via CoverM. The bacterial taxonomic labels and abundance in NHP gut metagenomes were analysed via MetaPhlAn4 [50] using the database ‘mpa_vOct22_CHOCOPhlAnSGB_202 212’.

### (g) Identification and phylogenetic comparison of crAss-like phage from nonhuman primates and humans

crAss-like phage, which were first detected in metagenomes, are prevalent in the human gut and regarded as human faecal indicators [51]. To explore the distribution of crAss-like phage in NHP guts, we identified crAss-like phage in the IGPC following the method described in [51]. Amino acid sequences of phage were aligned via BLASTP against the polymerase (UGP_018) and terminase (UGP_092) of the prototypical crAssphage (p-crAssphage, NC_024711.1) [52]. Phage genomes were considered to indicate crAss-like phage if they met the following criteria: (1) an *E*-value of less than 1*E*−05 against either the p-crAssphage polymerase or terminase; (2) an alignment length of at least 350 bp; and (3) a total genome length of greater than 70 kb. Based on the amino acid sequences of phage, a rooted species tree was inferred by OrthoFinder [53]. Weighted UniFrac distances were calculated using the UniFrac function in the phyloseq package in R with the option ‘weighted = TRUE’.

We downloaded 922 crAss-like phage genomes from humans reported in previous studies [51,54]. To compare differences in crAss-like phage between humans and NHPs, multiple sequence alignments of their terminase protein sequences (UGP_092) were implemented via MAFFT [55]. Based on the multiple sequence alignment, a phylogenetic tree was reconstructed via FastTree [56] and visualized via GraPhlAn [57]. Pairwise distances among crAss-like phage were calculated based on the terminase phylogenetic tree using the cophenetic.phylo function in the ape package (https://github.com/emmanuelparadis/ape) in R.

### (h) Statistical analysis of data

Shannon indices of gut phage and bacteria in NHPs were calculated via the diversity function of the vegan package (https://github.com/vegandevs/vegan) in R. The association between phage and bacterial Shannon diversity was examined using a linear mixed-effects model (LMM) with host species and data source study as random intercepts, and bacterial diversity as a fixed effect. We used an LMM with host clade and dietary niche as fixed effects, and host species and data source study as random intercepts to assess the effects of NHP clade and dietary niche, and restricted these analyses to wild individuals only (*n* = 286). *Post hoc* pairwise comparisons were conducted using estimated marginal means (EMMs), with false discovery rate (FDR) correction. To assess the effects of NHP lifestyle, we used samples of each NHP species with different lifestyles from a single study. A linear model (LM) was used to assess the effect of lifestyle for each NHP species. For lifestyle factors with three levels, *post hoc* pairwise comparisons were conducted using EMMs, with FDR correction; no adjustment was applied for two-level factors. The phage taxa and AMG categories with significant abundance differences were analysed via MaAsLin2 [58] with the option: max_significance = 0.05. For these MaAsLin2 analyses, we included host clade and dietary niche as fixed effects, with host species and data source study as random effects for the clade and diet analysis, and included lifestyle as the fixed effect for the lifestyle analysis. Principal coordinate analysis (PCoA) based on Bray–Curtis distance and Jaccard distance was performed using the vegan package in R. Permutational multivariate analysis of variance (PERMANOVA) with 999 permutations was performed using the adonis2 function of the vegan package in R. Multivariate homogeneity of group dispersions was tested using the betadisper function in the vegan package in R to assess the significance of dispersion differences.

A complete description of all methods, including software tools, versions and command-line parameters, is provided in the supplementary material.

## 3. Results

### (a) Construction of a nonredundant gut phage catalogue of nonhuman primates

To characterize the gut phageomes of NHPs, we performed the reconstruction of gut phage genomes from 454 metagenomic samples of 25 NHP species (figure 1A). The phylogenetic tree of 25 NHP species was constructed based on the topology reported in [59], adapted to include only the species present in our study. We assembled all gut metagenomes of NHPs and obtained 13 572 446 contigs with length >1500 bp. After identification via VirSorter2 and DeepVirFinder, 428 968 contigs were regarded as putative phage contigs. CheckV analysis identified 69 920 (16.3%), 344 729 (80.4%), 8302 (1.9%), 5821 (1.4%) and 196 (0.05%) phage contigs estimated as not determined, low quality, medium quality, high quality and complete, respectively. After clustering phage genomes classified as complete, high quality, and medium quality, we obtained 11 389 nonredundant genomes, namely the IGPC. The length of 74.2% of the phage genomes in the IGPC ranged from 1500 to 60 000 bp (figure 1B). In IGPC, 311 contigs (2.7%) were longer than 200 kb. Additionally, 28.30, 20.26 and 9.97% of the long contigs were assigned to *Suoliviridae*_like, *Kyanoviridae* and *Tybeckvirinae*_like, respectively (supplementary material, figure S1A); 45.66, 14.15 and 10.29% were predicted to infect Firmicutes, Proteobacteria and Bacteroidetes, respectively (supplementary material, figure S1B); 9.32, 6.75 and 6.11% were predicted to infect *Lactobacillus*, *Clostridium* and *Prevotella*, respectively (supplementary material, figure S1C). The total relative abundance of these long contigs accounted for 0.00033 to 45.37% of the total phage abundance, with an average abundance of 2.11%. Among the phage genomes in the IGPC, 171 (1.5%), 5064 (44.5%) and 6154 (54.0%) were classified as complete, high quality, and medium quality, respectively (figure 1C). The lifestyles of 11 154 phage genomes (97.9%) in the IGPC were predicted via PhaTYP; 4302 (38.6%) were temperate phage and 6852 (61.4%) were virulent phage (figure 1D).

In the IGPC, 1353 phage genomes (11.9%) were classified into 194 types at the genus level via PhaGenus. Among these classified genera, the most abundant genus was *Fipvunavirus* (28.8%), followed by *Takahashivirus* (12.4%) and *Audreyjarvisvirus* (5.4%; figure 1E). A total of 10 002 phage genomes (87.8%) in the IGPC were classified at the family or family-like level via PhaGCN2. ‘Family-like’ means that the virus is probably closely related to the reference family or belongs to the same order but a different family [34]. Among these classified families, 1068 phage genomes (10.7%) were classified into *Tybeckvirinae*_like, 926 (9.3%) into *Salasmaviridae* and 698 (7.0%) into *Salasmaviridae*_like (figure 1F).

The prokaryotic hosts of 11 257 phage genomes (98.8%) in the IGPC were classified into 2784 species via CHERRY. The 2784 species belonged to two superkingdoms, 36 phyla, 65 classes, 144 orders, 295 families and 902 genera (figure 1G). The most prevalent phylum was Firmicutes (*n* = 5456), followed by Proteobacteria (*n* = 1665), Bacteroidetes (*n* = 1174), Actinobacteria (*n* = 698) and Crenarchaeota (*n* = 589) (supplementary material, figure S2). The most prevalent family was Clostridiaceae (*n* = 852), followed by Lachnospiraceae (*n* = 656), Ruminococcaceae (*n* = 544), Lactobacillaceae (*n* = 514) and Thermoproteaceae (*n* = 477). Among the phage genomes of *Uroviricota*, most were virulent phage (63.1%), and their prokaryotic hosts were mainly Firmicutes (50.4%) and Proteobacteria (13.5%; figure 1H). For *Preplasmiviricota*, 58.4% of the phage were virulent, and their prokaryotic hosts were mainly Firmicutes (38.8%) and Proteobacteria (16.9%).

Compared with those in the NCBI viral reference genomes, IMG/VR v4 database, GPD, GVD and MGV databases, 10.0, 68.5, 56.7, 42.5 and 56.2% respectivelyof the phage genomes in the IGPC had ANIs >95% (figure 1I). In addition, 61.5% of phage genomes in the IGPC had an ANI > 95% compared with the integrated human phage set including the GPD, GVD and MGV databases.

### (b) Abundance characteristic of phage in the integrated gut phage catalogue

The abundance of phage in the IGPC was quantified in all NHP metagenomes. A total of 5682 phage genomes were shared among the four clades of NHPs (figure 2A). There were 1, 20, 1036 and 48 phage genomes that were detected only in the guts of Lemuriformes, Platyrrhini, Cercopithecidae and Hominoidea, respectively. There were 568 phage (5.0%) that were present in more than 90% of the samples, defined as core phage (figure 2B). There were 2883 phage (25.3%) that were present in 50–90% of the samples, defined as common phage. There were 7938 phage (69.7%) that were present in fewer than 50% of the samples, defined as low-prevalence phage.

**Figure 2.**
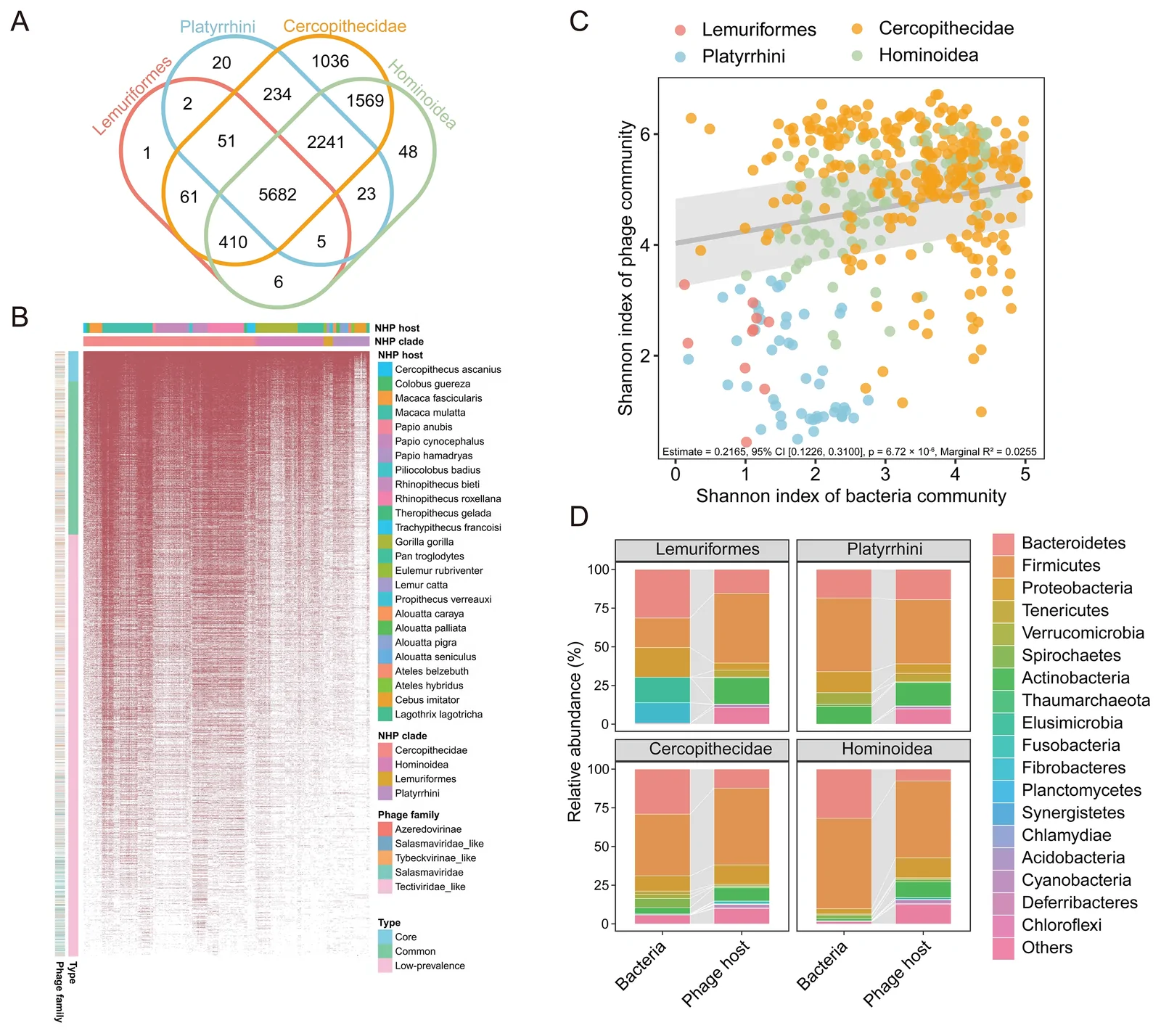
Abundances of integrated gut phage catalogue phage in nonhuman primate (NHP) guts. (A) Venn diagram of the number of gut phage identified in Lemuriformes, Platyrrhini, Cercopithecidae and Hominoidea. (B) Distributions of core (present in >90% of samples), common (50–90%) and low-prevalence (<50%) phage in all NHP guts. Red in the heatmap denotes the existence of phage in the sample. (C) Correlation of Shannon index between bacterial and phage communities. The grey line and shaded region represent the fitted regression line and its 95% confidence interval, respectively. Fixed-effect estimate = 0.2165, 95% CI = [0.1226, 0.3100], marginal *R*^2^ = 0.0255 and *p* = 6.72 × 10^−6^. (D) Phylum abundance of bacteria and phage hosts in the guts of Lemuriformes, Platyrrhini, Cercopithecidae and Hominoidea.

Shannon indices of the respective communities in the guts of NHPs were calculated to explore the association between gut phage and bacterial diversities. An LMM revealed a significant positive association between bacterial and phage Shannon diversity (fixed effect estimate = 0.2165, 95% CI = [0.1226, 0.3100], marginal *R*^2^ = 0.0255, *p* = 6.72 × 10^−6^; figure 2C). Additionally, Bacteroidetes and Firmicutes were the primary phyla shared by the phage hosts and the gut bacteria across the four clades of NHPs (figure 2D).

### (c) Responses of gut phage in nonhuman primates to host clade and dietary niche

To analyse the influence of different clades and dietary niches on gut phage of NHPs, we compared the diversity and function of the IGPC phage in the guts of all wild NHPs. PCoA plots of the abundance of gut phage based on Bray‒Curtis distance (figure 3A; PERMANOVA, host clade: *R*^2^ = 0.1363, *p* = 0.001; dietary niche: *R*^2^ = 0.0255, *p* = 0.001) and Jaccard distance (figure 3B; PERMANOVA, host clade: *R*^2^ = 0.2059, *p* = 0.001; dietary niche: *R*^2^ = 0.0445, *p* = 0.001) revealed that these samples were more distinctly clustered according to NHP clades. Beta dispersion analysis identified significant dispersion differences among host clades (Bray–Curtis: *F*_3,282_ = 88.481, *p* < 0.05, *R*² = 0.485; Jaccard: *F*_3,282_ = 49.554, *p* < 0.05, *R*^2^ = 0.345), but no such differences between dietary groups (Bray–Curtis: *F*_1,284_ = 1.11, *p* = 0.2923, *R*² = 0.004; Jaccard: *F*_1,284_ = 0.905, *p* = 0.342, *R*^2^ = 0.003). PCoA plots showed that samples from different host clades formed distinct clusters with well separated centroids, although beta dispersion analysis revealed significant differences in within-group dispersion among clades. The tree generated by the distribution of gut phage based on Euclidean distance using a presence/absence matrix showed that Cercopithecidae and Hominoidea species clustered together, as did Lemuriformes and Platyrrhini (figure 3C).

**Figure 3.**
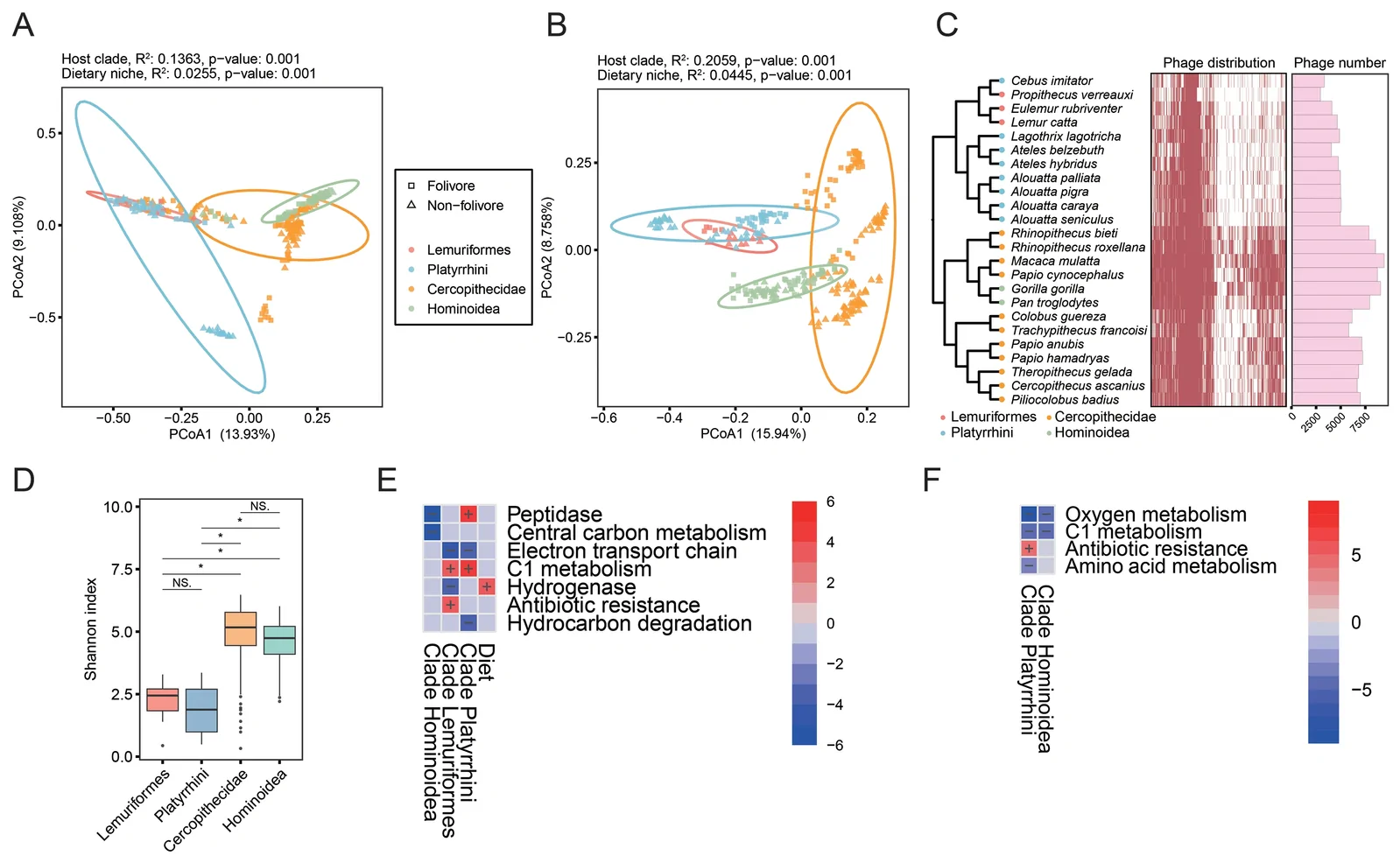
Gut phage diversity and auxiliary metabolic genes (AMGs) across wild nonhuman primates (NHPs) with different clades and dietary niches. (A) Principal coordinate analysis (PCoA) plot based on Bray‒Curtis distance of integrated gut phage catalogue (IGPC) abundance in all wild NHP guts. (B) PCoA plot based on Jaccard distance of IGPC abundance in all wild NHP guts. (C) Tree generated by distribution of gut phage according to Euclidean distance via presence/absence standardization. (D) Shannon index comparisons of gut phage in wild Lemuriformes, Platyrrhini, Cercopithecidae and Hominoidea. *p < 0.05; NS., not significant. (E) Comparisons of AMG category of temperate phage across wild NHP clades and dietary niches via MaAsLin2. +, significant enrichment in nonfolivore for diet and non-Cercopithecidae for clade; −, significant enrichment in folivore for diet and Cercopithecidae for clade. (F) Comparisons of AMG category of virulent phage across wild NHP clades and dietary niches via MaAsLin2. +, significant enrichment in nonfolivore for diet and non-Cercopithecidae for clade; −, enrichment in folivore for diet and Cercopithecidae for clade.

We used LMMs to evaluate the effects of NHP clade and dietary niche on the Shannon diversity and abundance of gut phage. The main-effects model revealed a significant effect of host clade (Type III Wald test, *χ*^2^ = 18.9810, d.f. = 3, *p* = 0.00028), while dietary niche showed no significant effect (*χ*^2^ = 2.0463, d.f. = 1, *p* = 0.1526). *Post hoc* pairwise comparisons revealed that Cercopithecidae and Hominoidea had significantly higher Shannon diversity than Lemuriformes and Platyrrhini (EMMs, adjusted *p* < 0.05; figure 3D). For the abundance of temperate and virulent phage, the main-effects model revealed no significant effects of host clade (Type III Wald test, temperate: *χ*² = 5.7693, *p* = 0.1234; virulent: *χ*² = 2.3683, *p* = 0.4996) and dietary niche (temperate: *χ*² = 0.0222, *p* = 0.8816; virulent: *χ*² = 0.1057, *p* = 0.7451), indicating that the abundance of phage with different lifestyles did not vary significantly with host clade or dietary niche. Compared with Cercopithecidae, most phage families exhibited significantly lower abundance in Platyrrhini and Lemuriformes, while only a few families showed significant differences between folivores and nonfolivores (supplementary material, figure S3A).

The AMGs of the IGPC phage were functionally annotated, and the abundance of AMGs in the guts of all wild NHPs was calculated. Information system and amino acid metabolism were the main categories in temperate and virulent phage (supplementary material, figure S3B). AMGs associated with the electron transport chain, hydrocarbon degradation and hydrogenase were detected only in temperate phage, whereas AMGs associated with adenosylcobalamin synthesis, aerobic corrin ring synthesis, flagella structure, oxygen metabolism and short-chain fatty acid (SCFA) and alcohol conversion were detected only in virulent phage. For temperate phage, hydrogenase function was significantly enriched in nonfolivores (figure 3E). Compared with Cercopithecidae, there were significant differences in seven AMG functions in the other three clades, including those involved in peptidase, central carbon metabolism and electron transport chain. For virulent phage, no significant difference in AMG function was detected between folivores and nonfolivores (figure 3F). Compared with Platyrrhini, oxygen metabolism, C1 metabolism and amino acid metabolism were significantly enriched in Cercopithecidae. Compared with Hominoidea, oxygen metabolism and C1 metabolism were significantly enriched in Cercopithecidae.

### (d) Influence of different nonhuman primate lifestyles on gut phage

To evaluate the influence of different NHP lifestyles on gut phage, we selected five species of NHPs living in captive, semiwild or wild environments. All samples of each NHP species, including *Rhinopithecus bieti* [21], *Trachypithecus francoisi* [23], *Rhinopithecus roxellana* [24], *Pan troglodytes* [18] and *Gorilla gorilla* [18], were derived from a single study. PCoA plots based on Bray‒Curtis distance of the abundance of gut phage revealed that these samples were significantly clustered according to different lifestyles in all NHPs (PERMANOVA, lifestyle: *R*^2^ = 0.2610‒0.3896, *p* = 0.001; figure 4A). Gut phage Shannon diversity was generally significantly higher in captive or semiwild individuals than in wild individuals across most NHP species (EMMs, *p* < 0.05; figure 4B). Captive *P*. *troglodytes*, semiwild *R*. *bieti* and semiwild *R*. *roxellana* exhibited significantly greater abundances of temperate phage than their wild counterparts did (EMMs, *p* < 0.05; figure 4C). For virulent phage, wild *R*. *bieti* had significantly higher abundance than semiwild individuals, captive *R*. *roxellana* had significantly higher abundance than wild individuals, and captive *T*. *francoisi* had significantly higher abundance than both semiwild and wild individuals (EMMs, *p* < 0.05).

**Figure 4.**
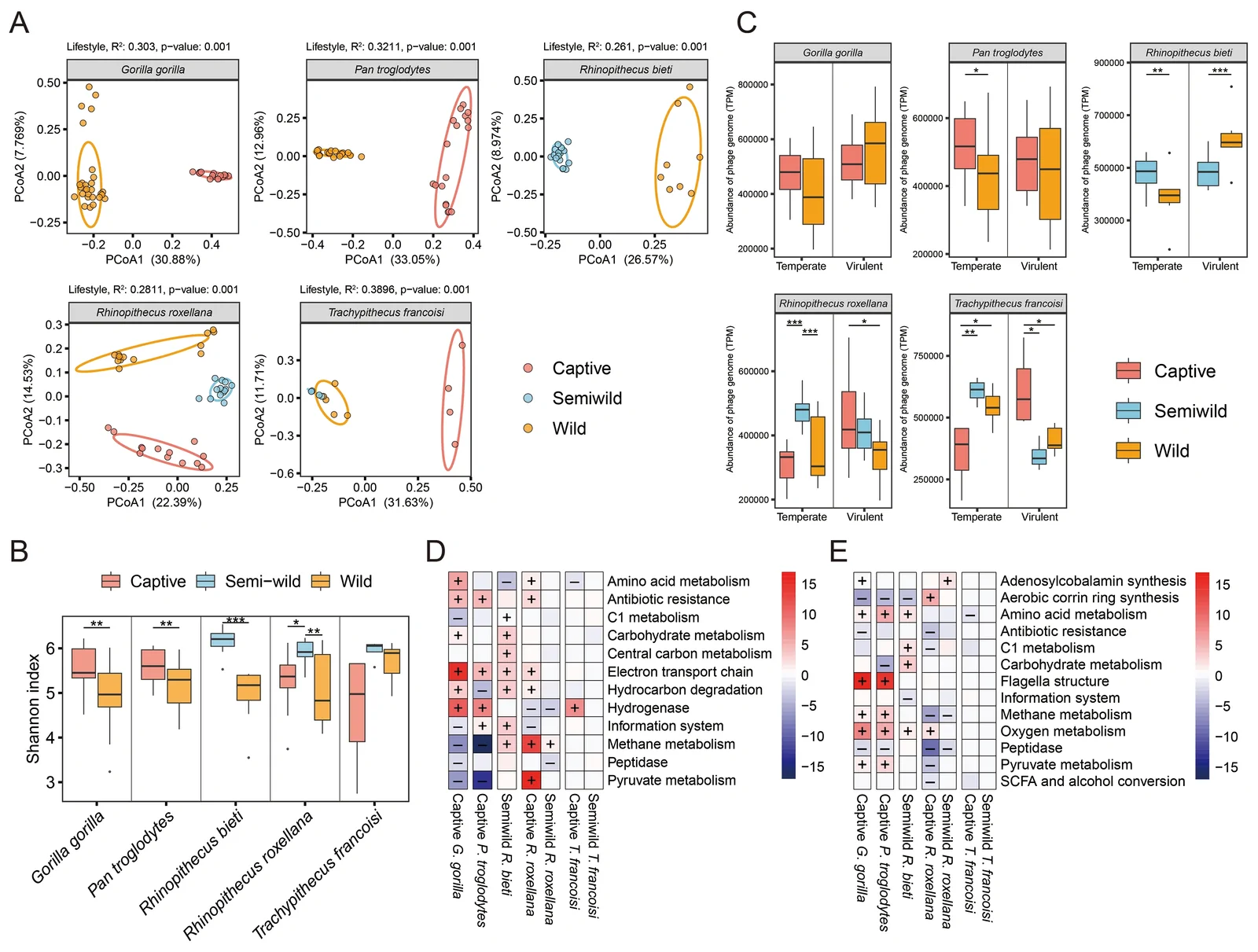
Gut phage diversity and auxiliary metabolic genes across *Gorilla gorilla, Pan troglodytes, Rhinopithecus bieti, Rhinopithecus roxellana* and *Trachypithecus francoisi*, with different lifestyles. (A) Principal coordinate analysis plot based on Bray–Curtis distance of phage abundance in the guts of nonhuman primates (NHPs) with different lifestyles. (B) Shannon index comparisons of gut phage in NHPs with different lifestyles. *p < 0.05, **p < 0.01, ***p < 0.001. (C) Abundance comparisons of virulent and temperate phage in the guts of NHPs. *p < 0.05, **p < 0.01, ***p < 0.001. TPM, transcripts per million. (D) Comparisons of AMG category of temperate phage in NHPs with different lifestyles via MaAsLin2. +, enrichment in captive or semiwild individuals; −, enrichment in wild individuals. (E) Comparisons of AMG category of virulent phage in NHPs with different lifestyles via MaAsLin2. +, significant enrichment in captive or semiwild individuals; −, significant enrichment in wild individuals. SCFA, short-chain fatty acid.

In temperate phage, most functional categories of AMGs, such as antibiotic resistance, carbohydrate metabolism and electron transport chain (figure 4D), were significantly enriched in the captive or semiwild individuals. In virulent phage, adenosylcobalamin synthesis, flagella structure and oxygen metabolism were significantly enriched only in captive or semiwild individuals (figure 4E).

### (e) Abundance of integrated gut phage catalogue phage in the guts of humans from industrialized and nonindustrialized countries

To explore the similarity of gut phage between NHPs and humans, we calculated the abundance of IGPC phage in 80 gut metagenomes of humans from industrialized and nonindustrialized countries. A total of 8946 phage (78.5%) in the IGPC were detected in the guts of humans, 284 of which were detected only in the guts of humans from industrialized countries and 2538 of which were detected only in the guts of humans from nonindustrialized countries (figure 5A). The prevalence of IGPC phage in the guts of humans from nonindustrialized countries was significantly greater than that in the guts of humans from industrialized countries (EMMs, *p* < 0.001; figure 5B). The PCoA plot based on the Jaccard distance of the abundance of gut phage in humans and all wild NHPs revealed that human samples were closely clustered with Cercopithecidae and Hominoidea samples (figure 5C). The PCoA plot based on the Jaccard distance of the gut phage abundance showed that the human samples from nonindustrialized countries were more closely clustered with the samples from *G*. *gorilla* and *P*. *troglodytes* than with those from industrialized countries (figure 5D).

**Figure 5.**
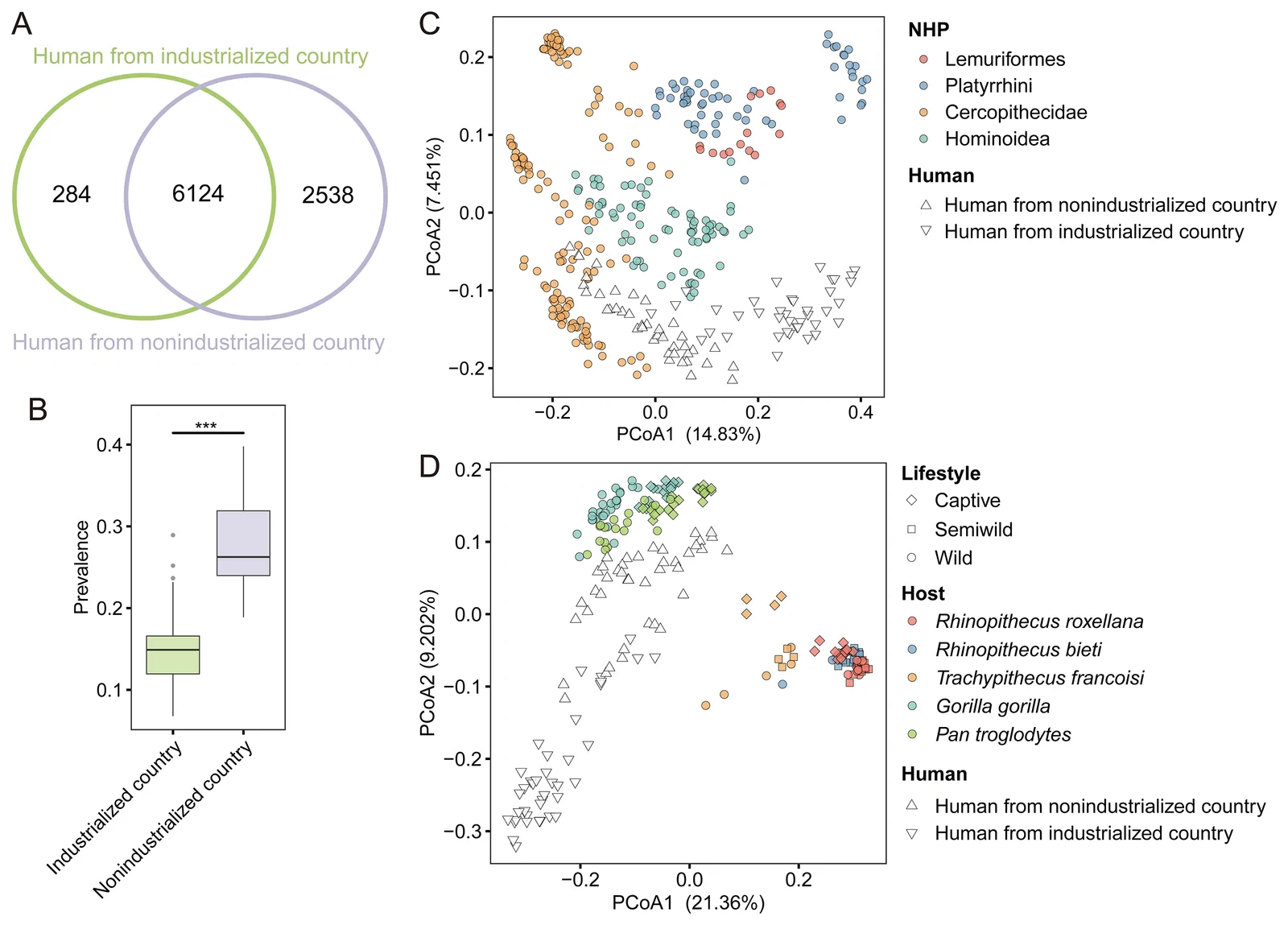
Comparative analysis of gut phage communities between nonhuman primates and humans. (A) Venn diagram of the number of integrated gut phage catalogue (IGPC) gut phage in humans from nonindustrialized and industrialized countries. (B) Prevalence of IGPC phage in guts of humans from nonindustrialized and industrialized countries. ***p < 0.001. (C) Principal coordinate analysis (PCoA) plot based on Jaccard distance of gut phage abundance in humans, Lemuriformes, Platyrrhini, Cercopithecidae and Hominoidea. (D) PCoA plot based on Jaccard distance of gut phage abundance in humans and *Rhinopithecus bieti, Trachypithecus francoisi, Rhinopithecus roxellana, Pan troglodytes* and *Gorilla gorilla*, which have different lifestyles. Point shape indicates host lifestyle; point colour indicates host species.

### (f) Comparison of crAss-like phage in the guts of nonhuman primates and humans

In the IGPC, 567 crAss-like phage were identified. Among these, 177 phage had only the polymerase-encoding gene (UGP_018) detected, 235 had only the terminase-coding gene (UGP_092) detected and 155 had both genes detected. Among these crAss-like phage, 249, 172 and 120 belonged to Steigviridae, Suoliviridae and Intestiviridae, respectively (figure 6A). The prokaryotic hosts of 185, 170 and 86 crAss-like phage belonged to Bacteroidetes, Firmicutes and Proteobacteria, respectively. PERMANOVA on the weighted UniFrac distance matrix revealed a significant effect of host clade and dietary niche on the phylogenetic composition of crAss-like phage (clade: *R*² = 0.0970, *p* = 0.001; diet: *R*² = 0.0294, *p* = 0.001; supplementary material, figure S4). The main-effects model revealed the significant effects of host clade (Type III Wald test, *χ*^2^ = 32.0435, d.f. = 3, *p* = 5.124 × 10⁻⁷) and dietary niche (*χ*^2^ = 4.2117, d.f. = 1, *p* = 0.0401). *Post hoc* pairwise comparisons showed that crAss-like phage abundance was significantly higher in both wild Cercopithecidae and Hominoidea than in wild Lemuriformes and Platyrrhini (EMMs, adjusted *p* < 0.01; figure 6B). The prevalence of crAss-like phage in the guts of captive *R*. *roxellana* and *T*. *francoisi* was significantly greater than that in the guts of their wild counterparts (EMMs, *p* < 0.01; figure 6C). Conversely, the prevalence of crAss-like phage in the guts of wild *G*. *gorilla* and *P*. *troglodytes* was significantly greater than that in the guts of captive individuals (EMMs, *p* < 0.05). The prevalence of crAss-like phage from the IGPC in the guts of humans from nonindustrialized countries was significantly greater than that in the guts of humans from industrialized countries (EMMs, *p* < 0.001; figure 6D).

**Figure 6.**
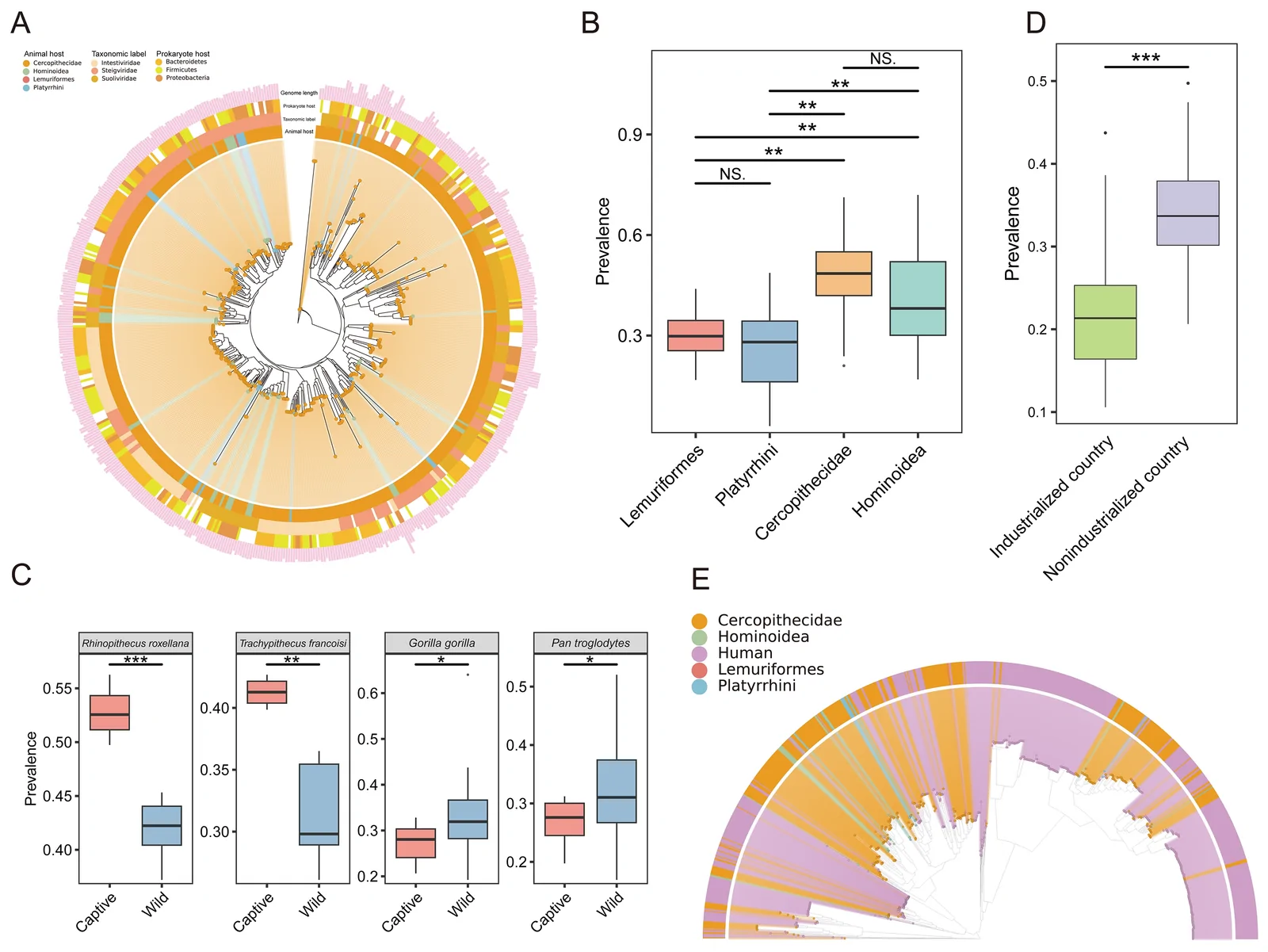
Comparison of crAss-like phage between humans and nonhuman primates (NHPs). (A) Phylogenetic tree of crAss-like phage from NHPs. (B) Comparison of prevalence of crAss-like phage in guts of wild Lemuriformes, Platyrrhini, Cercopithecidae and Hominoidea. **, adjusted *p* < 0.01; NS., not significant. (C) Comparison of prevalence of crAss-like phage in guts of captive and wild *Rhinopithecus roxellana, Trachypithecus francoisi, Gorilla gorilla* and *Pan troglodytes* *p < 0.05, **p < 0.01, ***p < 0.001. (D) Comparison of prevalence of crAss-like phage in human samples from nonindustrialized and industrialized countries. ***p < 0.001. (E) Phylogenic tree of genes encoding terminase (UGP_092) of crAss-like phage from NHPs and humans.

The phylogenetic tree of these terminase-encoding genes in crAss-like phage revealed that human crAss-like phage formed a distinct and monophyletic clade, clearly separated from NHP-derived crAss-like phage (figure 6E). Only a few NHP crAss-like phage were interspersed within the human clade, indicating limited cross-species transmission. PERMANOVA revealed a highly significant effect of host origin (human versus NHP) on the phylogenetic distance matrix (*R*^2^ = 0.0972, *p* = 0.001), indicating that human-derived crAss-like phage were phylogenetically distinct from those derived from NHPs.

## 4. Discussion

In this study, we analysed 454 gut metagenomes from 25 NHP species and constructed a nonredundant gut phage catalogue comprising 11 389 phage genomes. Compared with several public virome databases, approximately 40% of phage in the IGPC are novel, significantly expanding the known diversity of phage genomes in the NHP guts.

Host phylogeny primarily shapes the frequency of specific microbial populations, whereas host dietary type mediates the assembly of the bacterial community [60]. Hale *et al.* [61] reported that the gut microbiome of colobine monkeys was strongly influenced by host diet, moderately by host genus and weakly by host species. Amato *et al.* [13] demonstrated that the gut microbial composition and function of NHPs were more strongly influenced by host clades (at superfamily level or higher taxonomic ranks) than by host dietary niche. In our study, we also found that the gut phage diversity of NHPs was more strongly influenced by host clades than by host dietary niche. Therefore, the strength of the phylogenetic effect of host on the gut microbiome or phageome may depend on the host taxonomic scale considered, with higher taxonomic ranks often exhibiting more pronounced phylogenetic effects [62]. Additionally, we found that the dietary effect on the gut phageome appeared to be secondary to host phylogeny. The omnivorous diet of NHPs contains high fibre and carbohydrate, which might result in a weaker difference between folivores and nonfolivores, unlike the pronounced differences between herbivores and carnivores [63].

We detected more AMG categories in virulent phage than in temperate phage. A previous study reported that virulent phage from water, particles and sediment exhibited greater AMG diversity [64]. This may result from the wider host spectrum and higher infection frequency of virulent phage, which grant them access to a more extensive gene pool [64]. In contrast, the frequent horizontal gene transfer in temperate phage may lead to the homogenization of their AMGs. We found that the enrichment of hydrogenase in the gut phage of nonfolivores may reflect adaptation to host gut environments with lower fibre-derived hydrogen production [65]. The absence of significant AMG differences in virulent phage between folivores and nonfolivores, as well as the fewer functional contrasts across NHP host clades, may imply the conservation of AMG function among different NHP hosts, possibly owing to a broader host range of virulent phage and their higher infection frequency [64]. Most AMG categories were enriched in most captive individuals across both temperate and virulent phage, implying that captivity selected for phage carrying broad metabolic gene repertoires through lifestyle changes and environmental shifts.

NHPs and humans share not only a close genetic relationship but also similar gut microbiome compositions. NHPs in captivity develop a gut microbial composition similar to that of humans [66]. Many studies have shown that the composition of the human gut microbiome is influenced by two dietary types: a Westernized diet (typical of industrialized countries) and a non-Westernized diet (typical of nonindustrialized countries) [67]. We found that there was an obvious difference in the IGPC phage composition in the guts of humans from industrialized and nonindustrialized countries, suggesting human dietary changes might drive the evolution of gut phage composition. In particular, the human lifestyle of industrialized countries significantly drives divergence of the gut phageome from that of NHPs. In addition, the phage composition of IGPC in human guts was more similar to that in Hominoidea, probably owing to the closer evolutionary relationship between Hominoidea NHPs and humans.

Our results show that crAss-like phage have also been widely found in the guts of NHPs, but exhibit distinct evolutionary differences between NHPs and humans. The results indicated an ancient common origin followed by evolutionary divergence of crAss-like phage between NHPs and humans [68]. We found that the prevalence of crAss-like phage in samples from captive *R*. *roxellana* and *T*. *francoisi* was significantly greater than that in samples from their wild counterparts. This difference could be attributable to the horizontal transfer of crAss-like phage through frequent contact with humans in captive environments. In contrast, the higher prevalence of crAss-like phage in wild great apes compared with their captive counterparts may reflect the retention of ancient phage lineages shared with humans, which are disrupted in captivity by dietary shifts, antibiotic use and altered social structures. These results further suggest that the gut phage responses to animal lifestyle differ across NHP species. The phylogenetic analysis of crAss-like phage terminase revealed a host-specific clustering pattern, with human crAss-like phage forming a distinct monophyletic clade clearly separated from those from NHPs. These findings highlight that primate crAss-like phage exhibited strong host specificity, probably driven by long-term coevolutionary dynamics and barriers to cross-host transmission.

Additionally, several limitations of our study must be considered when interpreting these findings. The reference-dependent quality assessment used by CheckV may misclassify novel and well assembled phage as low-quality genomes. Excluding such genomes prioritizes reliability but probably underestimates true phage diversity. Future studies incorporating reference-free quality metrics or expanded databases can improve the capture of this hidden diversity. Predictions of phage lifestyle and host are influenced by genome completeness; short contigs often lack hallmark genes or host-specific signatures, reducing prediction accuracy. Although this limitation is inherent to metagenome-based studies, our conclusions—drawn from population-level trends across hundreds of samples—remain robust. TPM is commonly used in metagenomic normalization, but it provides only relative abundance, which limits the interpretation of absolute abundance differences. Several studies have demonstrated that absolute abundance offers greater informational depth than relative abundance and can correct findings that rely solely on relative abundance data [69]. Therefore, future studies employing absolute quantification methods will be valuable to validate and extend our findings.

Additionally, detailed metadata on individual age and sex could not be reliably obtained from the 13 public datasets used in this meta-analysis, and thus were not included in our analysis. Previous studies in captive *Macacamulatta* [8,70], wild *M. mulatta* [71] and wild *Macaca assamensis* [72] found no significant differences in gut microbial diversity or core taxa related to sex or age among non-infant individuals. Similarly, no sex-based differences were detected among adult wild western lowland gorillas (*Gorilla gorilla gorilla*) [73]. Nonetheless, the lack of detailed metadata is a limitation of this study, and future studies with systematic collection of age and sex information are needed to further confirm their potential roles.

In conclusion, we present an NHP gut phage catalogue comprising 11 389 nonredundant phage genomes. Evaluating phage genomes from metagenomes overcomes the difficulty of laboratory phage culture and isolation. These results reveal how host clade, dietary niche and lifestyle shape gut phage diversity, composition and metabolic function in NHPs. Overall, in this study, these phage genomic resources greatly advance a better understanding of the function and evolution of gut phage of primates.

## Supplementary Material



## Data Availability

The IGPC sequences generated in this study have been deposited in the Figshare database under accession code [74] and in the CNGB Sequence Archive (CNSA) of the China National GeneBank DataBase (CNGBdb) under accession number CNP0006761, accessible at https://db.cngb.org/data_resources/project/CNP0006761. All bioinformatics analysis pipelines, source data tables and R scripts supporting this study are available from Figshare database under accession code [74]. Supplementary material is available online [75].
