# Supplementary material for: An integrated gut phage catalogue of nonhuman primates reveals functional adaptation to host ecology and evolutionary link to humans
Source: Proc Biol Sci. 2026 Nov 4;293(2082):20260827. doi: 10.1098/rspb.2026.0827 (PMC13730807; doi:10.1098/rspb.2026.0827)
Supplement: Supplementary file 1 [file rspb.2026.0827.zip › rspb.2026.0827/rspb20260827_si_001.pdf]

Electronic Supplementary Material

An integrated gut phage catalog of nonhuman primates reveals functional adaptation to host ecology and evolutionary link to humans

Shengzhi Yang[1,†], Lei Jin[2,†], Wenwen Deng[2,3,†], Hongtao Xiao[1], Yongfang Yao[1], Meng Xie[1], Huailiang Xu[1,*], Likou Zou[2,*]

[1] College of Life Science, Sichuan Agricultural University, Ya'an, Sichuan, China

[2] College of Resources, Sichuan Agricultural University, Chengdu, Sichuan, China

[3] Key Laboratory of State Forestry and Grassland Administration (SFGA) on Conservation Biology of Rare Animals in the Giant Panda National Park, The China Conservation and Research Center for the Giant Panda (CCRCGP), Chengdu, Sichuan, China

Journal: Proceedings of the Royal Society B: Biological Sciences

DOI: 10.1098/rspb.2026.0827

Table S1 The NHP information collected from metagenomes in this study

Host lifestyle	Host diet	Host clade	Host scientific name	Number of metagenomes	References
Captive	Folivore	Cercopithecidae	*Rhinopithecus roxellana*	22	[1, 2]
			Trachypithecus francoisi	4	[3]
		Hominoidea	*Gorilla gorilla*	15	[4]
	Nonfolivore	Cercopithecidae	*Macaca fascicularis*	20	[5]
			Macaca mulatta	56	[1, 6, 7]
		Hominoidea	*Pan troglodytes*	18	[4]
Semi-wild	Folivore	Cercopithecidae	*Rhinopithecus bieti*	16	[8]
			Rhinopithecus roxellana	13	[2]
			Trachypithecus francoisi	4	[3]
Wild	Folivore	Cercopithecidae	*Colobus guereza*	5	[9]
			Piliocolobus badius	5	[9]
			Rhinopithecus bieti	8	[8]
			Rhinopithecus roxellana	23	[1, 2]
			Theropithecus gelada	5	[9]
			Trachypithecus francoisi	5	[3]
		Hominoidea	*Gorilla gorilla*	52	[4, 9, 10]
		Lemuriformes	*Propithecus verreauxi*	5	[9]
		Platyrrhini	*Alouatta caraya*	5	[9]
			Alouatta palliata	6	[9]
			Alouatta pigra	8	[9]
			Alouatta seniculus	5	[9]
	Nonfolivore	Platyrrhini	*Ateles belzebuth*	5	[9]
			Ateles hybridus	5	[9]
		Cercopithecidae	*Cercopithecus ascanius*	5	[9]

			Macaca mulatta	24	[11]
			Papio anubis	5	[9]
			Papio cynocephalus	48	[12]
			Papio hamadryas	5	[9]
		Hominoidea	*Pan troglodytes*	23	[4, 9]
		Lemuriformes	*Eulemur rubriventer*	5	[9]
			Lemur catta	5	[9]
		Platyrrhini	*Cebus imitator*	19	[13]
			Lagothrix lagotricha	5	[9]

Reference

1. Wang X, Wang Z, Pan H, et al. Captivity influences the gut microbiome of *Rhinopithecus roxellana*. *Front Microbiol* 2021;**12**:763022. https://doi.org/10.3389/fmicb.2021.763022

2. Zhang D, Hu Q, Zhou Y, et al. Multi-omic profiling reveals distinct gut microbial and metabolic landscapes in golden snub-nosed monkeys under contrasting conservation strategies. *npj Biofilms Microbiomes* 2025;**11**:209. https://doi.org/10.1038/s41522-025-00836-1

3. Sun Y, Yu Y, Wu A, et al. The composition and function of the gut microbiota of Francois' langurs (*Trachypithecus francoisi*) depend on the environment and diet. *Front Microbiol* 2023;**14**:1269492. https://doi.org/10.3389/fmicb.2023.1269492

4. Campbell TP, Sun X, Patel VH, et al. The microbiome and resistome of chimpanzees, gorillas, and humans across host lifestyle and geography. *ISME J* 2020;**14**:1584–1599. https://doi.org/10.1038/s41396-020-0634-2

5. Li X, Liang S, Xia Z, et al. Establishment of a *Macaca fascicularis* gut microbiome gene catalog and comparison with the human, pig, and mouse gut microbiomes. *GigaScience* 2018;**7**:giy100. https://doi.org/10.1093/gigascience/giy100

6. Rhoades NS, Cinco IR, Hendrickson SM, et al. Taxonomic and functional shifts in the perinatal gut microbiome of rhesus macaques. *Microbiol Spectrum* 2022;**10**:e0081422. https://doi.org/10.1128/spectrum.00814-22

7. Yang S, Liu Y, Yang N, et al. The gut microbiome and antibiotic resistome of chronic diarrhea rhesus macaques (*Macaca mulatta*) and its similarity to the human gut microbiome. *Microbiome* 2022;**10**:29. https://doi.org/10.1186/s40168-021-01218-3

8. Xia W, Liu G, Wang D, et al. Functional convergence of Yunnan snub-nosed monkey and bamboo-eating panda gut microbiomes revealing the driving by dietary flexibility on mammal gut microbiome. *Comput Struct Biotechnol J* 2022;**20**:685–699. https://doi.org/10.1016/j.csbj.2022.01.011

9. Amato KR, G Sanders J, Song SJ, et al. Evolutionary trends in host physiology outweigh dietary niche in structuring primate gut microbiomes. *ISME J* 2019;**13**:576–587. https://doi.org/10.1038/s41396-018-0175-0

10. Hicks AL, Lee KJ, Couto-Rodriguez M, et al. Gut microbiomes of wild great apes fluctuate seasonally in response to diet. *Nat Commun* 2018;**9**:1786. https://doi.org/10.1038/s41467-018-04204-w

11. Li D, Xia W, Cui X, et al. The putatively high-altitude adaptation of macaque monkeys: Evidence from the fecal metabolome and gut microbiome. *Evol Appl* 2023;**16**:1708–1720. https://doi.org/10.1111/eva.13595

12. Tung J, Barreiro LB, Burns MB, et al. Social networks predict gut microbiome composition in wild baboons. *eLife* 2015;**4**:e05224. https://doi.org/10.7554/eLife.05224

13. Orkin JD, Campos FA, Myers MS, et al. Seasonality of the gut microbiota of free-ranging white-faced capuchins in a tropical dry forest. *ISME J* 2019;**13**:183–196. https://doi.org/10.1038/s41396-018-0256-0

Table S2 The sample information in this study

Sample_id	Lifestyle	Age	Sex	Host scientific name	Clade
Alouatta_caraya_1	Wild	Unavailable	Unavailable	*Alouatta caraya*	Platyrrhini
Alouatta_caraya_2	Wild	Unavailable	Unavailable	*Alouatta caraya*	Platyrrhini
Alouatta_caraya_3	Wild	Unavailable	Unavailable	*Alouatta caraya*	Platyrrhini
Alouatta_caraya_4	Wild	Unavailable	Unavailable	*Alouatta caraya*	Platyrrhini
Alouatta_caraya_5	Wild	Unavailable	Unavailable	*Alouatta caraya*	Platyrrhini
Alouatta_palliata_1	Wild	Unavailable	Unavailable	*Alouatta palliata*	Platyrrhini
Alouatta_palliata_2	Wild	Unavailable	Unavailable	*Alouatta palliata*	Platyrrhini
Alouatta_palliata_3	Wild	Unavailable	Unavailable	*Alouatta palliata*	Platyrrhini
Alouatta_palliata_4	Wild	Unavailable	Unavailable	*Alouatta palliata*	Platyrrhini
Alouatta_palliata_5	Wild	Unavailable	Unavailable	*Alouatta palliata*	Platyrrhini
Alouatta_palliata_6	Wild	Unavailable	Unavailable	*Alouatta palliata*	Platyrrhini
Alouatta_pigra_1	Wild	Unavailable	Unavailable	*Alouatta pigra*	Platyrrhini
Alouatta_pigra_2	Wild	Unavailable	Unavailable	*Alouatta pigra*	Platyrrhini
Alouatta_pigra_3	Wild	Unavailable	Unavailable	*Alouatta pigra*	Platyrrhini
Alouatta_pigra_4	Wild	Unavailable	Unavailable	*Alouatta pigra*	Platyrrhini
Alouatta_pigra_5	Wild	Unavailable	Unavailable	*Alouatta pigra*	Platyrrhini
Alouatta_pigra_6	Wild	Unavailable	Unavailable	*Alouatta pigra*	Platyrrhini
Alouatta_pigra_7	Wild	Unavailable	Unavailable	*Alouatta pigra*	Platyrrhini
Alouatta_pigra_8	Wild	Unavailable	Unavailable	*Alouatta pigra*	Platyrrhini
Alouatta_seniculus_1	Wild	Unavailable	Unavailable	*Alouatta seniculus*	Platyrrhini
Alouatta_seniculus_2	Wild	Unavailable	Unavailable	*Alouatta seniculus*	Platyrrhini
Alouatta_seniculus_3	Wild	Unavailable	Unavailable	*Alouatta seniculus*	Platyrrhini
Alouatta_seniculus_4	Wild	Unavailable	Unavailable	*Alouatta seniculus*	Platyrrhini
Alouatta_seniculus_5	Wild	Unavailable	Unavailable	*Alouatta seniculus*	Platyrrhini
Ateles_belzebuth_1	Wild	Unavailable	Unavailable	*Ateles belzebuth*	Platyrrhini
Ateles_belzebuth_2	Wild	Unavailable	Unavailable	*Ateles belzebuth*	Platyrrhini
Ateles_belzebuth_3	Wild	Unavailable	Unavailable	*Ateles belzebuth*	Platyrrhini
Ateles_belzebuth_4	Wild	Unavailable	Unavailable	*Ateles belzebuth*	Platyrrhini
Ateles_belzebuth_5	Wild	Unavailable	Unavailable	*Ateles belzebuth*	Platyrrhini

Ateles_hybridus_1	Wild	Unavailable	Unavailable	*Ateles hybridus*	Platyrrhini
Ateles_hybridus_2	Wild	Unavailable	Unavailable	*Ateles hybridus*	Platyrrhini
Ateles_hybridus_3	Wild	Unavailable	Unavailable	*Ateles hybridus*	Platyrrhini
Ateles_hybridus_4	Wild	Unavailable	Unavailable	*Ateles hybridus*	Platyrrhini
Ateles_hybridus_5	Wild	Unavailable	Unavailable	*Ateles hybridus*	Platyrrhini
Cebus_imitator_1	Wild	Adult	Female	*Cebus imitator*	Platyrrhini
Cebus_imitator_2	Wild	Adult	Female	*Cebus imitator*	Platyrrhini
Cebus_imitator_3	Wild	Adult	Male	*Cebus imitator*	Platyrrhini
Cebus_imitator_4	Wild	Adult	Male	*Cebus imitator*	Platyrrhini
Cebus_imitator_5	Wild	Adult	Male	*Cebus imitator*	Platyrrhini
Cebus_imitator_6	Wild	Adult	Female	*Cebus imitator*	Platyrrhini
Cebus_imitator_7	Wild	Adult	Female	*Cebus imitator*	Platyrrhini
Cebus_imitator_8	Wild	Adult	Male	*Cebus imitator*	Platyrrhini
Cebus_imitator_9	Wild	Adult	Female	*Cebus imitator*	Platyrrhini
Cebus_imitator_10	Wild	Adult	Female	*Cebus imitator*	Platyrrhini
Cebus_imitator_11	Wild	Adult	Male	*Cebus imitator*	Platyrrhini
Cebus_imitator_12	Wild	Adult	Male	*Cebus imitator*	Platyrrhini
Cebus_imitator_13	Wild	Adult	Female	*Cebus imitator*	Platyrrhini
Cebus_imitator_14	Wild	Adult	Male	*Cebus imitator*	Platyrrhini
Cebus_imitator_15	Wild	Adult	Female	*Cebus imitator*	Platyrrhini
Cebus_imitator_16	Wild	Adult	Female	*Cebus imitator*	Platyrrhini
Cebus_imitator_17	Wild	Adult	Male	*Cebus imitator*	Platyrrhini
Cebus_imitator_18	Wild	Adult	Female	*Cebus imitator*	Platyrrhini
Cebus_imitator_19	Wild	Adult	Female	*Cebus imitator*	Platyrrhini
Cercopithecus_ascanius_1	Wild	Unavailable	Unavailable	*Cercopithecus ascanius*	Cercopithecidae
Cercopithecus_ascanius_2	Wild	Unavailable	Unavailable	*Cercopithecus ascanius*	Cercopithecidae
Cercopithecus_ascanius_3	Wild	Unavailable	Unavailable	*Cercopithecus ascanius*	Cercopithecidae
Cercopithecus_ascanius_4	Wild	Unavailable	Unavailable	*Cercopithecus ascanius*	Cercopithecidae
Cercopithecus_ascanius_5	Wild	Unavailable	Unavailable	*Cercopithecus ascanius*	Cercopithecidae
Colobus_guereza_1	Wild	Unavailable	Unavailable	*Colobus guereza*	Cercopithecidae
Colobus_guereza_2	Wild	Unavailable	Unavailable	*Colobus guereza*	Cercopithecidae
Colobus_guereza_3	Wild	Unavailable	Unavailable	*Colobus guereza*	Cercopithecidae

Colobus_guereza_4	Wild	Unavailable	Unavailable	*Colobus guereza*	Cercopithecidae
Colobus_guereza_5	Wild	Unavailable	Unavailable	*Colobus guereza*	Cercopithecidae
Eulemur_rubriventer_1	Wild	Unavailable	Unavailable	*Eulemur rubriventer*	Lemuriformes
Eulemur_rubriventer_2	Wild	Unavailable	Unavailable	*Eulemur rubriventer*	Lemuriformes
Eulemur_rubriventer_3	Wild	Unavailable	Unavailable	*Eulemur rubriventer*	Lemuriformes
Eulemur_rubriventer_4	Wild	Unavailable	Unavailable	*Eulemur rubriventer*	Lemuriformes
Eulemur_rubriventer_5	Wild	Unavailable	Unavailable	*Eulemur rubriventer*	Lemuriformes
Gorilla_gorilla_1	Wild	Unavailable	Unavailable	*Gorilla gorilla*	Hominoidea
Gorilla_gorilla_2	Wild	Unavailable	Unavailable	*Gorilla gorilla*	Hominoidea
Gorilla_gorilla_3	Wild	Unavailable	Unavailable	*Gorilla gorilla*	Hominoidea
Gorilla_gorilla_4	Wild	Unavailable	Unavailable	*Gorilla gorilla*	Hominoidea
Gorilla_gorilla_5	Wild	Unavailable	Unavailable	*Gorilla gorilla*	Hominoidea
Gorilla_gorilla_6	Wild	Unavailable	Unavailable	*Gorilla gorilla*	Hominoidea
Gorilla_gorilla_7	Wild	Unavailable	Unavailable	*Gorilla gorilla*	Hominoidea
Gorilla_gorilla_8	Wild	Unavailable	Unavailable	*Gorilla gorilla*	Hominoidea
Gorilla_gorilla_9	Wild	Unavailable	Unavailable	*Gorilla gorilla*	Hominoidea
Gorilla_gorilla_10	Wild	Unavailable	Unavailable	*Gorilla gorilla*	Hominoidea
Gorilla_gorilla_11	Wild	Unavailable	Unavailable	*Gorilla gorilla*	Hominoidea
Gorilla_gorilla_12	Wild	Unavailable	Unavailable	*Gorilla gorilla*	Hominoidea
Gorilla_gorilla_13	Wild	Unavailable	Unavailable	*Gorilla gorilla*	Hominoidea
Gorilla_gorilla_14	Wild	Unavailable	Unavailable	*Gorilla gorilla*	Hominoidea
Gorilla_gorilla_15	Wild	Unavailable	Unavailable	*Gorilla gorilla*	Hominoidea
Gorilla_gorilla_16	Wild	Unavailable	Unavailable	*Gorilla gorilla*	Hominoidea
Gorilla_gorilla_17	Wild	Unavailable	Unavailable	*Gorilla gorilla*	Hominoidea
Gorilla_gorilla_18	Wild	Unavailable	Unavailable	*Gorilla gorilla*	Hominoidea
Gorilla_gorilla_19	Wild	Unavailable	Unavailable	*Gorilla gorilla*	Hominoidea
Gorilla_gorilla_20	Wild	Unavailable	Unavailable	*Gorilla gorilla*	Hominoidea
Gorilla_gorilla_21	Wild	Unavailable	Unavailable	*Gorilla gorilla*	Hominoidea
Gorilla_gorilla_22	Wild	Unavailable	Unavailable	*Gorilla gorilla*	Hominoidea
Gorilla_gorilla_23	Wild	Unavailable	Unavailable	*Gorilla gorilla*	Hominoidea
Gorilla_gorilla_24	Wild	Unavailable	Unavailable	*Gorilla gorilla*	Hominoidea
Gorilla_gorilla_25	Wild	Unavailable	Unavailable	*Gorilla gorilla*	Hominoidea

Gorilla_gorilla_26	Wild	Unavailable	Unavailable	*Gorilla gorilla*	Hominoidea
Gorilla_gorilla_27	Wild	Unavailable	Unavailable	*Gorilla gorilla*	Hominoidea
Gorilla_gorilla_28	Wild	Unavailable	Unavailable	*Gorilla gorilla*	Hominoidea
Gorilla_gorilla_29	Captive	Unavailable	Unavailable	*Gorilla gorilla*	Hominoidea
Gorilla_gorilla_30	Captive	Unavailable	Unavailable	*Gorilla gorilla*	Hominoidea
Gorilla_gorilla_31	Captive	Unavailable	Unavailable	*Gorilla gorilla*	Hominoidea
Gorilla_gorilla_32	Captive	Unavailable	Unavailable	*Gorilla gorilla*	Hominoidea
Gorilla_gorilla_33	Captive	Unavailable	Unavailable	*Gorilla gorilla*	Hominoidea
Gorilla_gorilla_34	Captive	Unavailable	Unavailable	*Gorilla gorilla*	Hominoidea
Gorilla_gorilla_35	Captive	Unavailable	Unavailable	*Gorilla gorilla*	Hominoidea
Gorilla_gorilla_36	Captive	Unavailable	Unavailable	*Gorilla gorilla*	Hominoidea
Gorilla_gorilla_37	Captive	Unavailable	Unavailable	*Gorilla gorilla*	Hominoidea
Gorilla_gorilla_38	Wild	Unavailable	Unavailable	*Gorilla gorilla*	Hominoidea
Gorilla_gorilla_39	Captive	Unavailable	Unavailable	*Gorilla gorilla*	Hominoidea
Gorilla_gorilla_40	Wild	Unavailable	Unavailable	*Gorilla gorilla*	Hominoidea
Gorilla_gorilla_41	Wild	Unavailable	Unavailable	*Gorilla gorilla*	Hominoidea
Gorilla_gorilla_42	Wild	Unavailable	Unavailable	*Gorilla gorilla*	Hominoidea
Gorilla_gorilla_43	Captive	Unavailable	Unavailable	*Gorilla gorilla*	Hominoidea
Gorilla_gorilla_44	Captive	Unavailable	Unavailable	*Gorilla gorilla*	Hominoidea
Gorilla_gorilla_45	Captive	Unavailable	Unavailable	*Gorilla gorilla*	Hominoidea
Gorilla_gorilla_46	Captive	Unavailable	Unavailable	*Gorilla gorilla*	Hominoidea
Gorilla_gorilla_47	Captive	Unavailable	Unavailable	*Gorilla gorilla*	Hominoidea
Gorilla_gorilla_48	Wild	Unavailable	Unavailable	*Gorilla gorilla*	Hominoidea
Gorilla_gorilla_49	Wild	Unavailable	Unavailable	*Gorilla gorilla*	Hominoidea
Gorilla_gorilla_50	Wild	Unavailable	Unavailable	*Gorilla gorilla*	Hominoidea
Gorilla_gorilla_51	Wild	Unavailable	Unavailable	*Gorilla gorilla*	Hominoidea
Gorilla_gorilla_52	Wild	Unavailable	Unavailable	*Gorilla gorilla*	Hominoidea
Gorilla_gorilla_53	Wild	Unavailable	Unavailable	*Gorilla gorilla*	Hominoidea
Gorilla_gorilla_54	Wild	Unavailable	Unavailable	*Gorilla gorilla*	Hominoidea
Gorilla_gorilla_55	Wild	Unavailable	Unavailable	*Gorilla gorilla*	Hominoidea
Gorilla_gorilla_56	Wild	Unavailable	Unavailable	*Gorilla gorilla*	Hominoidea
Gorilla_gorilla_57	Wild	Unavailable	Unavailable	*Gorilla gorilla*	Hominoidea

Gorilla_gorilla_58	Wild	Unavailable	Unavailable	*Gorilla gorilla*	Hominoidea
Gorilla_gorilla_59	Wild	Unavailable	Unavailable	*Gorilla gorilla*	Hominoidea
Gorilla_gorilla_60	Wild	Unavailable	Unavailable	*Gorilla gorilla*	Hominoidea
Gorilla_gorilla_61	Wild	Unavailable	Unavailable	*Gorilla gorilla*	Hominoidea
Gorilla_gorilla_62	Wild	Unavailable	Unavailable	*Gorilla gorilla*	Hominoidea
Gorilla_gorilla_63	Wild	Unavailable	Unavailable	*Gorilla gorilla*	Hominoidea
Gorilla_gorilla_64	Wild	Unavailable	Unavailable	*Gorilla gorilla*	Hominoidea
Gorilla_gorilla_65	Wild	Unavailable	Unavailable	*Gorilla gorilla*	Hominoidea
Gorilla_gorilla_66	Wild	Unavailable	Unavailable	*Gorilla gorilla*	Hominoidea
Gorilla_gorilla_67	Wild	Unavailable	Unavailable	*Gorilla gorilla*	Hominoidea
Lagothrix_lagotricha_1	Wild	Unavailable	Unavailable	*Lagothrix lagotricha*	Platyrrhini
Lagothrix_lagotricha_2	Wild	Unavailable	Unavailable	*Lagothrix lagotricha*	Platyrrhini
Lagothrix_lagotricha_3	Wild	Unavailable	Unavailable	*Lagothrix lagotricha*	Platyrrhini
Lagothrix_lagotricha_4	Wild	Unavailable	Unavailable	*Lagothrix lagotricha*	Platyrrhini
Lagothrix_lagotricha_5	Wild	Unavailable	Unavailable	*Lagothrix lagotricha*	Platyrrhini
Lemur_catta_1	Wild	Unavailable	Unavailable	*Lemur catta*	Lemuriformes
Lemur_catta_2	Wild	Unavailable	Unavailable	*Lemur catta*	Lemuriformes
Lemur_catta_3	Wild	Unavailable	Unavailable	*Lemur catta*	Lemuriformes
Lemur_catta_4	Wild	Unavailable	Unavailable	*Lemur catta*	Lemuriformes
Lemur_catta_5	Wild	Unavailable	Unavailable	*Lemur catta*	Lemuriformes
Macaca_fascicularis_1	Captive	Geratic	Unavailable	*Macaca fascicularis*	Cercopithecidae
Macaca_fascicularis_2	Captive	Geratic	Unavailable	*Macaca fascicularis*	Cercopithecidae
Macaca_fascicularis_3	Captive	Geratic	Unavailable	*Macaca fascicularis*	Cercopithecidae
Macaca_fascicularis_4	Captive	Geratic	Unavailable	*Macaca fascicularis*	Cercopithecidae
Macaca_fascicularis_5	Captive	Geratic	Unavailable	*Macaca fascicularis*	Cercopithecidae
Macaca_fascicularis_6	Captive	Geratic	Unavailable	*Macaca fascicularis*	Cercopithecidae
Macaca_fascicularis_7	Captive	Geratic	Unavailable	*Macaca fascicularis*	Cercopithecidae
Macaca_fascicularis_8	Captive	Geratic	Unavailable	*Macaca fascicularis*	Cercopithecidae
Macaca_fascicularis_9	Captive	Geratic	Unavailable	*Macaca fascicularis*	Cercopithecidae
Macaca_fascicularis_10	Captive	Geratic	Unavailable	*Macaca fascicularis*	Cercopithecidae
Macaca_fascicularis_11	Captive	Geratic	Unavailable	*Macaca fascicularis*	Cercopithecidae
Macaca_fascicularis_12	Captive	Geratic	Unavailable	*Macaca fascicularis*	Cercopithecidae

Macaca_fascicularis_13	Captive	Geratic	Unavailable	*Macaca fascicularis*	Cercopithecidae
Macaca_fascicularis_14	Captive	Geratic	Unavailable	*Macaca fascicularis*	Cercopithecidae
Macaca_fascicularis_15	Captive	Geratic	Unavailable	*Macaca fascicularis*	Cercopithecidae
Macaca_fascicularis_16	Captive	Geratic	Unavailable	*Macaca fascicularis*	Cercopithecidae
Macaca_fascicularis_17	Captive	Geratic	Unavailable	*Macaca fascicularis*	Cercopithecidae
Macaca_fascicularis_18	Captive	Geratic	Unavailable	*Macaca fascicularis*	Cercopithecidae
Macaca_fascicularis_19	Captive	Geratic	Unavailable	*Macaca fascicularis*	Cercopithecidae
Macaca_fascicularis_20	Captive	Geratic	Unavailable	*Macaca fascicularis*	Cercopithecidae
Macaca_mulatta_1	Captive	Unavailable	Unavailable	*Macaca mulatta*	Cercopithecidae
Macaca_mulatta_2	Captive	Unavailable	Unavailable	*Macaca mulatta*	Cercopithecidae
Macaca_mulatta_3	Captive	Unavailable	Unavailable	*Macaca mulatta*	Cercopithecidae
Macaca_mulatta_4	Captive	Unavailable	Unavailable	*Macaca mulatta*	Cercopithecidae
Macaca_mulatta_5	Captive	Unavailable	Unavailable	*Macaca mulatta*	Cercopithecidae
Macaca_mulatta_6	Captive	Unavailable	Unavailable	*Macaca mulatta*	Cercopithecidae
Macaca_mulatta_7	Captive	Unavailable	Unavailable	*Macaca mulatta*	Cercopithecidae
Macaca_mulatta_8	Captive	Unavailable	Unavailable	*Macaca mulatta*	Cercopithecidae
Macaca_mulatta_9	Captive	Unavailable	Unavailable	*Macaca mulatta*	Cercopithecidae
Macaca_mulatta_10	Captive	Unavailable	Unavailable	*Macaca mulatta*	Cercopithecidae
Macaca_mulatta_11	Captive	Unavailable	Unavailable	*Macaca mulatta*	Cercopithecidae
Macaca_mulatta_12	Captive	Unavailable	Unavailable	*Macaca mulatta*	Cercopithecidae
Macaca_mulatta_13	Captive	Unavailable	Unavailable	*Macaca mulatta*	Cercopithecidae
Macaca_mulatta_14	Captive	Unavailable	Unavailable	*Macaca mulatta*	Cercopithecidae
Macaca_mulatta_15	Captive	Unavailable	Unavailable	*Macaca mulatta*	Cercopithecidae
Macaca_mulatta_16	Captive	Unavailable	Unavailable	*Macaca mulatta*	Cercopithecidae
Macaca_mulatta_17	Captive	Unavailable	Unavailable	*Macaca mulatta*	Cercopithecidae
Macaca_mulatta_18	Captive	Unavailable	Unavailable	*Macaca mulatta*	Cercopithecidae
Macaca_mulatta_19	Captive	Unavailable	Unavailable	*Macaca mulatta*	Cercopithecidae
Macaca_mulatta_20	Captive	Unavailable	Unavailable	*Macaca mulatta*	Cercopithecidae
Macaca_mulatta_21	Captive	Unavailable	Unavailable	*Macaca mulatta*	Cercopithecidae
Macaca_mulatta_22	Captive	Unavailable	Unavailable	*Macaca mulatta*	Cercopithecidae
Macaca_mulatta_23	Captive	Unavailable	Unavailable	*Macaca mulatta*	Cercopithecidae
Macaca_mulatta_24	Captive	Unavailable	Unavailable	*Macaca mulatta*	Cercopithecidae

Macaca_mulatta_25	Captive	Unavailable	Unavailable	*Macaca mulatta*	Cercopithecidae
Macaca_mulatta_26	Captive	Unavailable	Unavailable	*Macaca mulatta*	Cercopithecidae
Macaca_mulatta_27	Captive	Unavailable	Unavailable	*Macaca mulatta*	Cercopithecidae
Macaca_mulatta_28	Captive	Unavailable	Unavailable	*Macaca mulatta*	Cercopithecidae
Macaca_mulatta_29	Captive	Unavailable	Unavailable	*Macaca mulatta*	Cercopithecidae
Macaca_mulatta_30	Captive	Unavailable	Unavailable	*Macaca mulatta*	Cercopithecidae
Macaca_mulatta_31	Captive	Unavailable	Unavailable	*Macaca mulatta*	Cercopithecidae
Macaca_mulatta_32	Captive	Unavailable	Unavailable	*Macaca mulatta*	Cercopithecidae
Macaca_mulatta_33	Captive	Unavailable	Unavailable	*Macaca mulatta*	Cercopithecidae
Macaca_mulatta_34	Captive	Unavailable	Unavailable	*Macaca mulatta*	Cercopithecidae
Macaca_mulatta_35	Captive	Unavailable	Unavailable	*Macaca mulatta*	Cercopithecidae
Macaca_mulatta_36	Captive	Unavailable	Unavailable	*Macaca mulatta*	Cercopithecidae
Macaca_mulatta_37	Captive	Unavailable	Unavailable	*Macaca mulatta*	Cercopithecidae
Macaca_mulatta_38	Captive	Unavailable	Unavailable	*Macaca mulatta*	Cercopithecidae
Macaca_mulatta_39	Captive	Unavailable	Unavailable	*Macaca mulatta*	Cercopithecidae
Macaca_mulatta_40	Captive	Subadult	Female	*Macaca mulatta*	Cercopithecidae
Macaca_mulatta_41	Captive	Subadult	Female	*Macaca mulatta*	Cercopithecidae
Macaca_mulatta_42	Captive	Geratic	Male	*Macaca mulatta*	Cercopithecidae
Macaca_mulatta_43	Captive	Subadult	Female	*Macaca mulatta*	Cercopithecidae
Macaca_mulatta_44	Captive	Geratic	Male	*Macaca mulatta*	Cercopithecidae
Macaca_mulatta_45	Captive	Geratic	Male	*Macaca mulatta*	Cercopithecidae
Macaca_mulatta_46	Captive	Subadult	Male	*Macaca mulatta*	Cercopithecidae
Macaca_mulatta_47	Captive	Subadult	Male	*Macaca mulatta*	Cercopithecidae
Macaca_mulatta_48	Captive	Subadult	Male	*Macaca mulatta*	Cercopithecidae
Macaca_mulatta_49	Captive	Geratic	Female	*Macaca mulatta*	Cercopithecidae
Macaca_mulatta_50	Captive	Geratic	Female	*Macaca mulatta*	Cercopithecidae
Macaca_mulatta_51	Captive	Geratic	Female	*Macaca mulatta*	Cercopithecidae
Macaca_mulatta_52	Captive	Adult	Male	*Macaca mulatta*	Cercopithecidae
Macaca_mulatta_53	Captive	Adult	Male	*Macaca mulatta*	Cercopithecidae
Macaca_mulatta_54	Captive	Adult	Female	*Macaca mulatta*	Cercopithecidae
Macaca_mulatta_55	Captive	Adult	Female	*Macaca mulatta*	Cercopithecidae
Macaca_mulatta_56	Captive	Adult	Female	*Macaca mulatta*	Cercopithecidae

Macaca_mulatta_57	Wild	Unavailable	Unavailable	*Macaca mulatta*	Cercopithecidae
Macaca_mulatta_58	Wild	Unavailable	Unavailable	*Macaca mulatta*	Cercopithecidae
Macaca_mulatta_59	Wild	Unavailable	Unavailable	*Macaca mulatta*	Cercopithecidae
Macaca_mulatta_60	Wild	Unavailable	Unavailable	*Macaca mulatta*	Cercopithecidae
Macaca_mulatta_61	Wild	Unavailable	Unavailable	*Macaca mulatta*	Cercopithecidae
Macaca_mulatta_62	Wild	Unavailable	Unavailable	*Macaca mulatta*	Cercopithecidae
Macaca_mulatta_63	Wild	Unavailable	Unavailable	*Macaca mulatta*	Cercopithecidae
Macaca_mulatta_64	Wild	Unavailable	Unavailable	*Macaca mulatta*	Cercopithecidae
Macaca_mulatta_65	Wild	Unavailable	Unavailable	*Macaca mulatta*	Cercopithecidae
Macaca_mulatta_66	Wild	Unavailable	Unavailable	*Macaca mulatta*	Cercopithecidae
Macaca_mulatta_67	Wild	Unavailable	Unavailable	*Macaca mulatta*	Cercopithecidae
Macaca_mulatta_68	Wild	Unavailable	Unavailable	*Macaca mulatta*	Cercopithecidae
Macaca_mulatta_69	Wild	Unavailable	Unavailable	*Macaca mulatta*	Cercopithecidae
Macaca_mulatta_70	Wild	Unavailable	Unavailable	*Macaca mulatta*	Cercopithecidae
Macaca_mulatta_71	Wild	Unavailable	Unavailable	*Macaca mulatta*	Cercopithecidae
Macaca_mulatta_72	Wild	Unavailable	Unavailable	*Macaca mulatta*	Cercopithecidae
Macaca_mulatta_73	Wild	Unavailable	Unavailable	*Macaca mulatta*	Cercopithecidae
Macaca_mulatta_74	Wild	Unavailable	Unavailable	*Macaca mulatta*	Cercopithecidae
Macaca_mulatta_75	Wild	Unavailable	Unavailable	*Macaca mulatta*	Cercopithecidae
Macaca_mulatta_76	Wild	Unavailable	Unavailable	*Macaca mulatta*	Cercopithecidae
Macaca_mulatta_77	Wild	Unavailable	Unavailable	*Macaca mulatta*	Cercopithecidae
Macaca_mulatta_78	Wild	Unavailable	Unavailable	*Macaca mulatta*	Cercopithecidae
Macaca_mulatta_79	Wild	Unavailable	Unavailable	*Macaca mulatta*	Cercopithecidae
Macaca_mulatta_80	Wild	Unavailable	Unavailable	*Macaca mulatta*	Cercopithecidae
Pan_troglodytes_1	Wild	Unavailable	Unavailable	*Pan troglodytes*	Hominoidea
Pan_troglodytes_2	Wild	Unavailable	Unavailable	*Pan troglodytes*	Hominoidea
Pan_troglodytes_3	Wild	Unavailable	Unavailable	*Pan troglodytes*	Hominoidea
Pan_troglodytes_4	Wild	Unavailable	Unavailable	*Pan troglodytes*	Hominoidea
Pan_troglodytes_5	Wild	Unavailable	Unavailable	*Pan troglodytes*	Hominoidea
Pan_troglodytes_6	Captive	Unavailable	Unavailable	*Pan troglodytes*	Hominoidea
Pan_troglodytes_7	Wild	Unavailable	Unavailable	*Pan troglodytes*	Hominoidea
Pan_troglodytes_8	Wild	Unavailable	Unavailable	*Pan troglodytes*	Hominoidea

Pan_troglodytes_9	Captive	Unavailable	Unavailable	*Pan troglodytes*	Hominoidea
Pan_troglodytes_10	Captive	Unavailable	Unavailable	*Pan troglodytes*	Hominoidea
Pan_troglodytes_11	Wild	Unavailable	Unavailable	*Pan troglodytes*	Hominoidea
Pan_troglodytes_12	Wild	Unavailable	Unavailable	*Pan troglodytes*	Hominoidea
Pan_troglodytes_13	Wild	Unavailable	Unavailable	*Pan troglodytes*	Hominoidea
Pan_troglodytes_14	Wild	Unavailable	Unavailable	*Pan troglodytes*	Hominoidea
Pan_troglodytes_15	Wild	Unavailable	Unavailable	*Pan troglodytes*	Hominoidea
Pan_troglodytes_16	Wild	Unavailable	Unavailable	*Pan troglodytes*	Hominoidea
Pan_troglodytes_17	Captive	Unavailable	Unavailable	*Pan troglodytes*	Hominoidea
Pan_troglodytes_18	Wild	Unavailable	Unavailable	*Pan troglodytes*	Hominoidea
Pan_troglodytes_19	Wild	Unavailable	Unavailable	*Pan troglodytes*	Hominoidea
Pan_troglodytes_20	Wild	Unavailable	Unavailable	*Pan troglodytes*	Hominoidea
Pan_troglodytes_21	Captive	Unavailable	Unavailable	*Pan troglodytes*	Hominoidea
Pan_troglodytes_22	Captive	Unavailable	Unavailable	*Pan troglodytes*	Hominoidea
Pan_troglodytes_23	Captive	Unavailable	Unavailable	*Pan troglodytes*	Hominoidea
Pan_troglodytes_24	Captive	Unavailable	Unavailable	*Pan troglodytes*	Hominoidea
Pan_troglodytes_25	Captive	Unavailable	Unavailable	*Pan troglodytes*	Hominoidea
Pan_troglodytes_26	Captive	Unavailable	Unavailable	*Pan troglodytes*	Hominoidea
Pan_troglodytes_27	Captive	Unavailable	Unavailable	*Pan troglodytes*	Hominoidea
Pan_troglodytes_28	Captive	Unavailable	Unavailable	*Pan troglodytes*	Hominoidea
Pan_troglodytes_29	Captive	Unavailable	Unavailable	*Pan troglodytes*	Hominoidea
Pan_troglodytes_30	Captive	Unavailable	Unavailable	*Pan troglodytes*	Hominoidea
Pan_troglodytes_31	Captive	Unavailable	Unavailable	*Pan troglodytes*	Hominoidea
Pan_troglodytes_32	Captive	Unavailable	Unavailable	*Pan troglodytes*	Hominoidea
Pan_troglodytes_33	Captive	Unavailable	Unavailable	*Pan troglodytes*	Hominoidea
Pan_troglodytes_34	Wild	Unavailable	Unavailable	*Pan troglodytes*	Hominoidea
Pan_troglodytes_35	Wild	Unavailable	Unavailable	*Pan troglodytes*	Hominoidea
Pan_troglodytes_36	Wild	Unavailable	Unavailable	*Pan troglodytes*	Hominoidea
Pan_troglodytes_37	Wild	Unavailable	Unavailable	*Pan troglodytes*	Hominoidea
Pan_troglodytes_38	Wild	Unavailable	Unavailable	*Pan troglodytes*	Hominoidea
Pan_troglodytes_39	Captive	Unavailable	Unavailable	*Pan troglodytes*	Hominoidea
Pan_troglodytes_40	Wild	Unavailable	Unavailable	*Pan troglodytes*	Hominoidea

Pan_troglodytes_41	Wild	Unavailable	Unavailable	*Pan troglodytes*	Hominoidea
Papio_anubis_1	Wild	Unavailable	Unavailable	*Papio anubis*	Cercopithecidae
Papio_anubis_2	Wild	Unavailable	Unavailable	*Papio anubis*	Cercopithecidae
Papio_anubis_3	Wild	Unavailable	Unavailable	*Papio anubis*	Cercopithecidae
Papio_anubis_4	Wild	Unavailable	Unavailable	*Papio anubis*	Cercopithecidae
Papio_anubis_5	Wild	Unavailable	Unavailable	*Papio anubis*	Cercopithecidae
Papio_cynocephalus_1	Wild	Adult	Male	*Papio cynocephalus*	Cercopithecidae
Papio_cynocephalus_2	Wild	Adult	Male	*Papio cynocephalus*	Cercopithecidae
Papio_cynocephalus_3	Wild	Adult	Male	*Papio cynocephalus*	Cercopithecidae
Papio_cynocephalus_4	Wild	Adult	Male	*Papio cynocephalus*	Cercopithecidae
Papio_cynocephalus_5	Wild	Adult	Male	*Papio cynocephalus*	Cercopithecidae
Papio_cynocephalus_6	Wild	Adult	Female	*Papio cynocephalus*	Cercopithecidae
Papio_cynocephalus_7	Wild	Adult	Female	*Papio cynocephalus*	Cercopithecidae
Papio_cynocephalus_8	Wild	Adult	Female	*Papio cynocephalus*	Cercopithecidae
Papio_cynocephalus_9	Wild	Adult	Female	*Papio cynocephalus*	Cercopithecidae
Papio_cynocephalus_10	Wild	Adult	Female	*Papio cynocephalus*	Cercopithecidae
Papio_cynocephalus_11	Wild	Adult	Female	*Papio cynocephalus*	Cercopithecidae
Papio_cynocephalus_12	Wild	Adult	Male	*Papio cynocephalus*	Cercopithecidae
Papio_cynocephalus_13	Wild	Adult	Male	*Papio cynocephalus*	Cercopithecidae
Papio_cynocephalus_14	Wild	Adult	Male	*Papio cynocephalus*	Cercopithecidae
Papio_cynocephalus_15	Wild	Adult	Male	*Papio cynocephalus*	Cercopithecidae
Papio_cynocephalus_16	Wild	Adult	Female	*Papio cynocephalus*	Cercopithecidae
Papio_cynocephalus_17	Wild	Adult	Female	*Papio cynocephalus*	Cercopithecidae
Papio_cynocephalus_18	Wild	Adult	Female	*Papio cynocephalus*	Cercopithecidae
Papio_cynocephalus_19	Wild	Adult	Female	*Papio cynocephalus*	Cercopithecidae
Papio_cynocephalus_20	Wild	Adult	Female	*Papio cynocephalus*	Cercopithecidae
Papio_cynocephalus_21	Wild	Adult	Female	*Papio cynocephalus*	Cercopithecidae
Papio_cynocephalus_22	Wild	Adult	Male	*Papio cynocephalus*	Cercopithecidae
Papio_cynocephalus_23	Wild	Adult	Male	*Papio cynocephalus*	Cercopithecidae
Papio_cynocephalus_24	Wild	Adult	Female	*Papio cynocephalus*	Cercopithecidae
Papio_cynocephalus_25	Wild	Adult	Male	*Papio cynocephalus*	Cercopithecidae
Papio_cynocephalus_26	Wild	Adult	Male	*Papio cynocephalus*	Cercopithecidae

Papio_cynocephalus_27	Wild	Adult	Female	*Papio cynocephalus*	Cercopithecidae
Papio_cynocephalus_28	Wild	Adult	Female	*Papio cynocephalus*	Cercopithecidae
Papio_cynocephalus_29	Wild	Adult	Female	*Papio cynocephalus*	Cercopithecidae
Papio_cynocephalus_30	Wild	Adult	Female	*Papio cynocephalus*	Cercopithecidae
Papio_cynocephalus_31	Wild	Adult	Male	*Papio cynocephalus*	Cercopithecidae
Papio_cynocephalus_32	Wild	Adult	Female	*Papio cynocephalus*	Cercopithecidae
Papio_cynocephalus_33	Wild	Adult	Female	*Papio cynocephalus*	Cercopithecidae
Papio_cynocephalus_34	Wild	Adult	Female	*Papio cynocephalus*	Cercopithecidae
Papio_cynocephalus_35	Wild	Adult	Female	*Papio cynocephalus*	Cercopithecidae
Papio_cynocephalus_36	Wild	Adult	Female	*Papio cynocephalus*	Cercopithecidae
Papio_cynocephalus_37	Wild	Adult	Female	*Papio cynocephalus*	Cercopithecidae
Papio_cynocephalus_38	Wild	Adult	Female	*Papio cynocephalus*	Cercopithecidae
Papio_cynocephalus_39	Wild	Adult	Female	*Papio cynocephalus*	Cercopithecidae
Papio_cynocephalus_40	Wild	Adult	Female	*Papio cynocephalus*	Cercopithecidae
Papio_cynocephalus_41	Wild	Adult	Male	*Papio cynocephalus*	Cercopithecidae
Papio_cynocephalus_42	Wild	Adult	Female	*Papio cynocephalus*	Cercopithecidae
Papio_cynocephalus_43	Wild	Adult	Female	*Papio cynocephalus*	Cercopithecidae
Papio_cynocephalus_44	Wild	Adult	Male	*Papio cynocephalus*	Cercopithecidae
Papio_cynocephalus_45	Wild	Adult	Female	*Papio cynocephalus*	Cercopithecidae
Papio_cynocephalus_46	Wild	Adult	Male	*Papio cynocephalus*	Cercopithecidae
Papio_cynocephalus_47	Wild	Adult	Female	*Papio cynocephalus*	Cercopithecidae
Papio_cynocephalus_48	Wild	Adult	Female	*Papio cynocephalus*	Cercopithecidae
Papio_hamadryas_1	Wild	Unavailable	Unavailable	*Papio hamadryas*	Cercopithecidae
Papio_hamadryas_2	Wild	Unavailable	Unavailable	*Papio hamadryas*	Cercopithecidae
Papio_hamadryas_3	Wild	Unavailable	Unavailable	*Papio hamadryas*	Cercopithecidae
Papio_hamadryas_4	Wild	Unavailable	Unavailable	*Papio hamadryas*	Cercopithecidae
Papio_hamadryas_5	Wild	Unavailable	Unavailable	*Papio hamadryas*	Cercopithecidae
Piliocolobus_badius_1	Wild	Unavailable	Unavailable	*Piliocolobus badius*	Cercopithecidae
Piliocolobus_badius_2	Wild	Unavailable	Unavailable	*Piliocolobus badius*	Cercopithecidae
Piliocolobus_badius_3	Wild	Unavailable	Unavailable	*Piliocolobus badius*	Cercopithecidae
Piliocolobus_badius_4	Wild	Unavailable	Unavailable	*Piliocolobus badius*	Cercopithecidae
Piliocolobus_badius_5	Wild	Unavailable	Unavailable	*Piliocolobus badius*	Cercopithecidae

Propithecus_verreauxi_1	Wild	Unavailable	Unavailable	*Propithecus verreauxi*	Lemuriformes
Propithecus_verreauxi_2	Wild	Unavailable	Unavailable	*Propithecus verreauxi*	Lemuriformes
Propithecus_verreauxi_3	Wild	Unavailable	Unavailable	*Propithecus verreauxi*	Lemuriformes
Propithecus_verreauxi_4	Wild	Unavailable	Unavailable	*Propithecus verreauxi*	Lemuriformes
Propithecus_verreauxi_5	Wild	Unavailable	Unavailable	*Propithecus verreauxi*	Lemuriformes
Rhinopithecus_bieti_1	Semiwild	Unavailable	Unavailable	*Rhinopithecus bieti*	Cercopithecidae
Rhinopithecus_bieti_2	Semiwild	Unavailable	Unavailable	*Rhinopithecus bieti*	Cercopithecidae
Rhinopithecus_bieti_3	Semiwild	Unavailable	Unavailable	*Rhinopithecus bieti*	Cercopithecidae
Rhinopithecus_bieti_4	Semiwild	Unavailable	Unavailable	*Rhinopithecus bieti*	Cercopithecidae
Rhinopithecus_bieti_5	Semiwild	Unavailable	Unavailable	*Rhinopithecus bieti*	Cercopithecidae
Rhinopithecus_bieti_6	Semiwild	Unavailable	Unavailable	*Rhinopithecus bieti*	Cercopithecidae
Rhinopithecus_bieti_7	Semiwild	Unavailable	Unavailable	*Rhinopithecus bieti*	Cercopithecidae
Rhinopithecus_bieti_8	Wild	Unavailable	Unavailable	*Rhinopithecus bieti*	Cercopithecidae
Rhinopithecus_bieti_9	Wild	Unavailable	Unavailable	*Rhinopithecus bieti*	Cercopithecidae
Rhinopithecus_bieti_10	Wild	Unavailable	Unavailable	*Rhinopithecus bieti*	Cercopithecidae
Rhinopithecus_bieti_11	Wild	Unavailable	Unavailable	*Rhinopithecus bieti*	Cercopithecidae
Rhinopithecus_bieti_12	Semiwild	Unavailable	Unavailable	*Rhinopithecus bieti*	Cercopithecidae
Rhinopithecus_bieti_13	Wild	Unavailable	Unavailable	*Rhinopithecus bieti*	Cercopithecidae
Rhinopithecus_bieti_14	Wild	Unavailable	Unavailable	*Rhinopithecus bieti*	Cercopithecidae
Rhinopithecus_bieti_15	Wild	Unavailable	Unavailable	*Rhinopithecus bieti*	Cercopithecidae
Rhinopithecus_bieti_16	Wild	Unavailable	Unavailable	*Rhinopithecus bieti*	Cercopithecidae
Rhinopithecus_bieti_17	Semiwild	Unavailable	Unavailable	*Rhinopithecus bieti*	Cercopithecidae
Rhinopithecus_bieti_18	Semiwild	Unavailable	Unavailable	*Rhinopithecus bieti*	Cercopithecidae
Rhinopithecus_bieti_19	Semiwild	Unavailable	Unavailable	*Rhinopithecus bieti*	Cercopithecidae
Rhinopithecus_bieti_20	Semiwild	Unavailable	Unavailable	*Rhinopithecus bieti*	Cercopithecidae
Rhinopithecus_bieti_21	Semiwild	Unavailable	Unavailable	*Rhinopithecus bieti*	Cercopithecidae
Rhinopithecus_bieti_22	Semiwild	Unavailable	Unavailable	*Rhinopithecus bieti*	Cercopithecidae
Rhinopithecus_bieti_23	Semiwild	Unavailable	Unavailable	*Rhinopithecus bieti*	Cercopithecidae
Rhinopithecus_bieti_24	Semiwild	Unavailable	Unavailable	*Rhinopithecus bieti*	Cercopithecidae
Rhinopithecus_roxellana_1	Wild	Unavailable	Unavailable	*Rhinopithecus roxellana*	Cercopithecidae
Rhinopithecus_roxellana_2	Captive	Unavailable	Unavailable	*Rhinopithecus roxellana*	Cercopithecidae
Rhinopithecus_roxellana_3	Captive	Unavailable	Unavailable	*Rhinopithecus roxellana*	Cercopithecidae

Rhinopithecus_roxellana_4	Captive	Unavailable	Unavailable	*Rhinopithecus roxellana*	Cercopithecidae
Rhinopithecus_roxellana_5	Captive	Unavailable	Unavailable	*Rhinopithecus roxellana*	Cercopithecidae
Rhinopithecus_roxellana_6	Captive	Unavailable	Unavailable	*Rhinopithecus roxellana*	Cercopithecidae
Rhinopithecus_roxellana_7	Captive	Unavailable	Unavailable	*Rhinopithecus roxellana*	Cercopithecidae
Rhinopithecus_roxellana_8	Captive	Unavailable	Unavailable	*Rhinopithecus roxellana*	Cercopithecidae
Rhinopithecus_roxellana_9	Wild	Unavailable	Unavailable	*Rhinopithecus roxellana*	Cercopithecidae
Rhinopithecus_roxellana_10	Wild	Unavailable	Unavailable	*Rhinopithecus roxellana*	Cercopithecidae
Rhinopithecus_roxellana_11	Wild	Unavailable	Unavailable	*Rhinopithecus roxellana*	Cercopithecidae
Rhinopithecus_roxellana_12	Wild	Unavailable	Unavailable	*Rhinopithecus roxellana*	Cercopithecidae
Rhinopithecus_roxellana_13	Wild	Unavailable	Unavailable	*Rhinopithecus roxellana*	Cercopithecidae
Rhinopithecus_roxellana_14	Wild	Unavailable	Unavailable	*Rhinopithecus roxellana*	Cercopithecidae
Rhinopithecus_roxellana_15	Wild	Unavailable	Unavailable	*Rhinopithecus roxellana*	Cercopithecidae
Rhinopithecus_roxellana_16	Wild	Unavailable	Unavailable	*Rhinopithecus roxellana*	Cercopithecidae
Rhinopithecus_roxellana_17	Captive	Unavailable	Unavailable	*Rhinopithecus roxellana*	Cercopithecidae
Rhinopithecus_roxellana_18	Captive	Unavailable	Unavailable	*Rhinopithecus roxellana*	Cercopithecidae
Rhinopithecus_roxellana_19	Wild	Unavailable	Unavailable	*Rhinopithecus roxellana*	Cercopithecidae
Rhinopithecus_roxellana_20	Captive	Unavailable	Unavailable	*Rhinopithecus roxellana*	Cercopithecidae
Rhinopithecus_roxellana_21	Captive	Unavailable	Unavailable	*Rhinopithecus roxellana*	Cercopithecidae
Rhinopithecus_roxellana_22	Wild	Unavailable	Unavailable	*Rhinopithecus roxellana*	Cercopithecidae
Rhinopithecus_roxellana_23	Captive	Unavailable	Unavailable	*Rhinopithecus roxellana*	Cercopithecidae
Rhinopithecus_roxellana_24	Captive	Unavailable	Unavailable	*Rhinopithecus roxellana*	Cercopithecidae
Rhinopithecus_roxellana_25	Captive	Unavailable	Unavailable	*Rhinopithecus roxellana*	Cercopithecidae
Rhinopithecus_roxellana_26	Captive	Unavailable	Unavailable	*Rhinopithecus roxellana*	Cercopithecidae
Rhinopithecus_roxellana_27	Wild	Unavailable	Unavailable	*Rhinopithecus roxellana*	Cercopithecidae
Rhinopithecus_roxellana_28	Captive	Unavailable	Unavailable	*Rhinopithecus roxellana*	Cercopithecidae
Rhinopithecus_roxellana_29	Captive	Unavailable	Unavailable	*Rhinopithecus roxellana*	Cercopithecidae
Rhinopithecus_roxellana_30	Captive	Unavailable	Unavailable	*Rhinopithecus roxellana*	Cercopithecidae
Rhinopithecus_roxellana_31	Wild	Unavailable	Unavailable	*Rhinopithecus roxellana*	Cercopithecidae
Rhinopithecus_roxellana_32	Wild	Unavailable	Unavailable	*Rhinopithecus roxellana*	Cercopithecidae
Rhinopithecus_roxellana_33	Wild	Unavailable	Unavailable	*Rhinopithecus roxellana*	Cercopithecidae
Rhinopithecus_roxellana_34	Wild	Unavailable	Unavailable	*Rhinopithecus roxellana*	Cercopithecidae
Rhinopithecus_roxellana_35	Wild	Unavailable	Unavailable	*Rhinopithecus roxellana*	Cercopithecidae

Rhinopithecus_roxellana_36	Semiwild	Unavailable	Unavailable	*Rhinopithecus roxellana*	Cercopithecidae
Rhinopithecus_roxellana_37	Semiwild	Unavailable	Unavailable	*Rhinopithecus roxellana*	Cercopithecidae
Rhinopithecus_roxellana_38	Semiwild	Unavailable	Unavailable	*Rhinopithecus roxellana*	Cercopithecidae
Rhinopithecus_roxellana_39	Semiwild	Unavailable	Unavailable	*Rhinopithecus roxellana*	Cercopithecidae
Rhinopithecus_roxellana_40	Semiwild	Unavailable	Unavailable	*Rhinopithecus roxellana*	Cercopithecidae
Rhinopithecus_roxellana_41	Semiwild	Unavailable	Unavailable	*Rhinopithecus roxellana*	Cercopithecidae
Rhinopithecus_roxellana_42	Captive	Unavailable	Unavailable	*Rhinopithecus roxellana*	Cercopithecidae
Rhinopithecus_roxellana_43	Captive	Unavailable	Unavailable	*Rhinopithecus roxellana*	Cercopithecidae
Rhinopithecus_roxellana_44	Captive	Unavailable	Unavailable	*Rhinopithecus roxellana*	Cercopithecidae
Rhinopithecus_roxellana_45	Semiwild	Unavailable	Unavailable	*Rhinopithecus roxellana*	Cercopithecidae
Rhinopithecus_roxellana_46	Captive	Unavailable	Unavailable	*Rhinopithecus roxellana*	Cercopithecidae
Rhinopithecus_roxellana_47	Wild	Unavailable	Unavailable	*Rhinopithecus roxellana*	Cercopithecidae
Rhinopithecus_roxellana_48	Wild	Unavailable	Unavailable	*Rhinopithecus roxellana*	Cercopithecidae
Rhinopithecus_roxellana_49	Semiwild	Unavailable	Unavailable	*Rhinopithecus roxellana*	Cercopithecidae
Rhinopithecus_roxellana_50	Semiwild	Unavailable	Unavailable	*Rhinopithecus roxellana*	Cercopithecidae
Rhinopithecus_roxellana_51	Semiwild	Unavailable	Unavailable	*Rhinopithecus roxellana*	Cercopithecidae
Rhinopithecus_roxellana_52	Semiwild	Unavailable	Unavailable	*Rhinopithecus roxellana*	Cercopithecidae
Rhinopithecus_roxellana_53	Semiwild	Unavailable	Unavailable	*Rhinopithecus roxellana*	Cercopithecidae
Rhinopithecus_roxellana_54	Semiwild	Unavailable	Unavailable	*Rhinopithecus roxellana*	Cercopithecidae
Rhinopithecus_roxellana_55	Wild	Unavailable	Unavailable	*Rhinopithecus roxellana*	Cercopithecidae
Rhinopithecus_roxellana_56	Wild	Unavailable	Unavailable	*Rhinopithecus roxellana*	Cercopithecidae
Rhinopithecus_roxellana_57	Wild	Unavailable	Unavailable	*Rhinopithecus roxellana*	Cercopithecidae
Rhinopithecus_roxellana_58	Wild	Unavailable	Unavailable	*Rhinopithecus roxellana*	Cercopithecidae
Theropithecus_gelada_1	Wild	Unavailable	Unavailable	*Theropithecus gelada*	Cercopithecidae
Theropithecus_gelada_2	Wild	Unavailable	Unavailable	*Theropithecus gelada*	Cercopithecidae
Theropithecus_gelada_3	Wild	Unavailable	Unavailable	*Theropithecus gelada*	Cercopithecidae
Theropithecus_gelada_4	Wild	Unavailable	Unavailable	*Theropithecus gelada*	Cercopithecidae
Theropithecus_gelada_5	Wild	Unavailable	Unavailable	*Theropithecus gelada*	Cercopithecidae
Trachypithecus_francoisi_1	Captive	Adult	Unavailable	*Trachypithecus francoisi*	Cercopithecidae
Trachypithecus_francoisi_2	Wild	Unavailable	Unavailable	*Trachypithecus francoisi*	Cercopithecidae
Trachypithecus_francoisi_3	Wild	Unavailable	Unavailable	*Trachypithecus francoisi*	Cercopithecidae
Trachypithecus_francoisi_4	Wild	Unavailable	Unavailable	*Trachypithecus francoisi*	Cercopithecidae

Trachypithecus_francoisi_5	Wild	Unavailable	Unavailable	*Trachypithecus francoisi*	Cercopithecidae
Trachypithecus_francoisi_6	Wild	Unavailable	Unavailable	*Trachypithecus francoisi*	Cercopithecidae
Trachypithecus_francoisi_7	Semiwild	Unavailable	Unavailable	*Trachypithecus francoisi*	Cercopithecidae
Trachypithecus_francoisi_8	Semiwild	Unavailable	Unavailable	*Trachypithecus francoisi*	Cercopithecidae
Trachypithecus_francoisi_9	Captive	Adult	Unavailable	*Trachypithecus francoisi*	Cercopithecidae
Trachypithecus_francoisi_10	Captive	Adult	Unavailable	*Trachypithecus francoisi*	Cercopithecidae
Trachypithecus_francoisi_11	Captive	Adult	Unavailable	*Trachypithecus francoisi*	Cercopithecidae
Trachypithecus_francoisi_12	Semiwild	Unavailable	Unavailable	*Trachypithecus francoisi*	Cercopithecidae
Trachypithecus_francoisi_13	Semiwild	Unavailable	Unavailable	*Trachypithecus francoisi*	Cercopithecidae

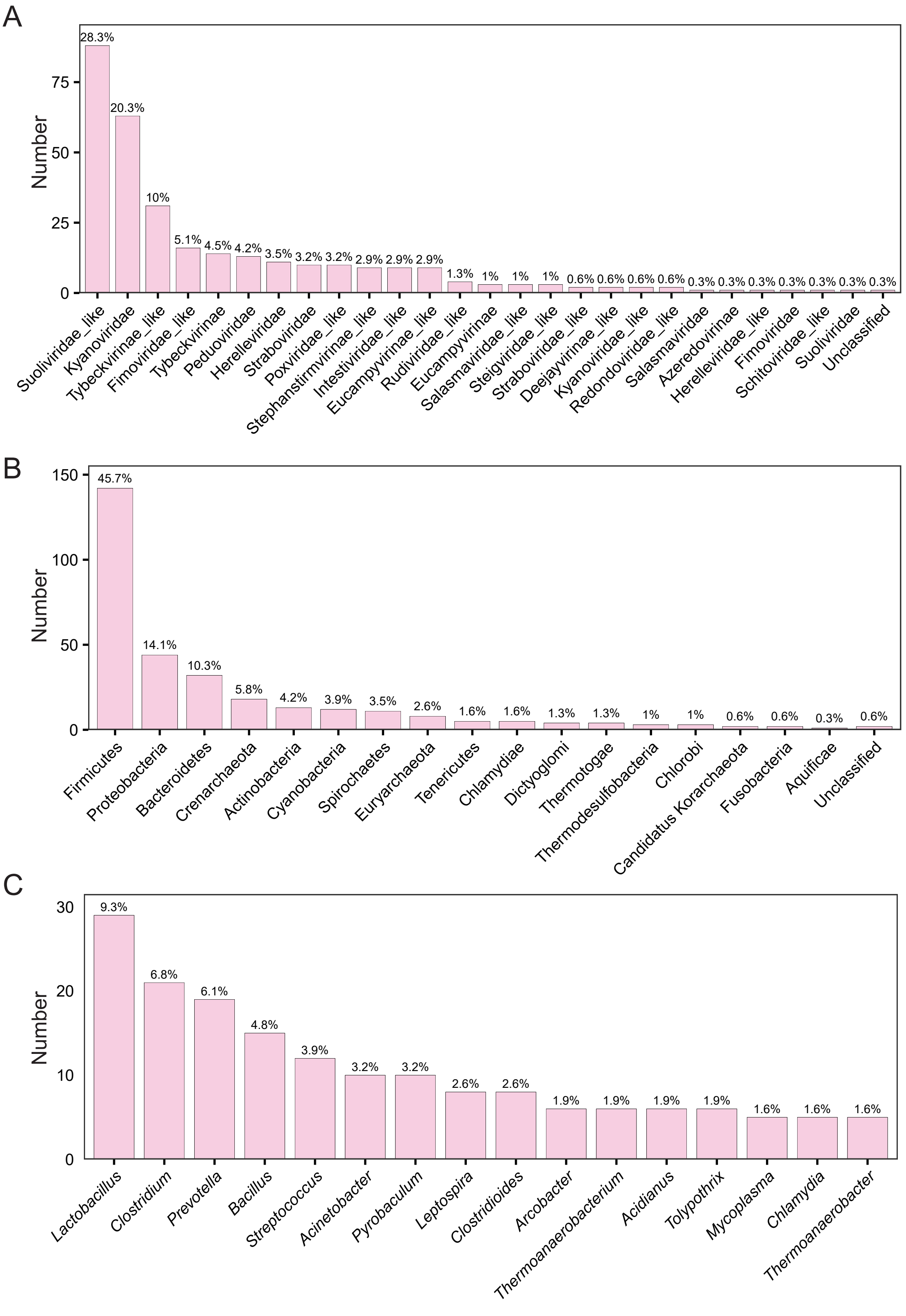

Figure S1 The genome characterization of phages more than 200kb in the IGPC. (A) The taxonomic families of phages; (B) The phyla of phage's hosts; (C) The main genera of phage's hosts.

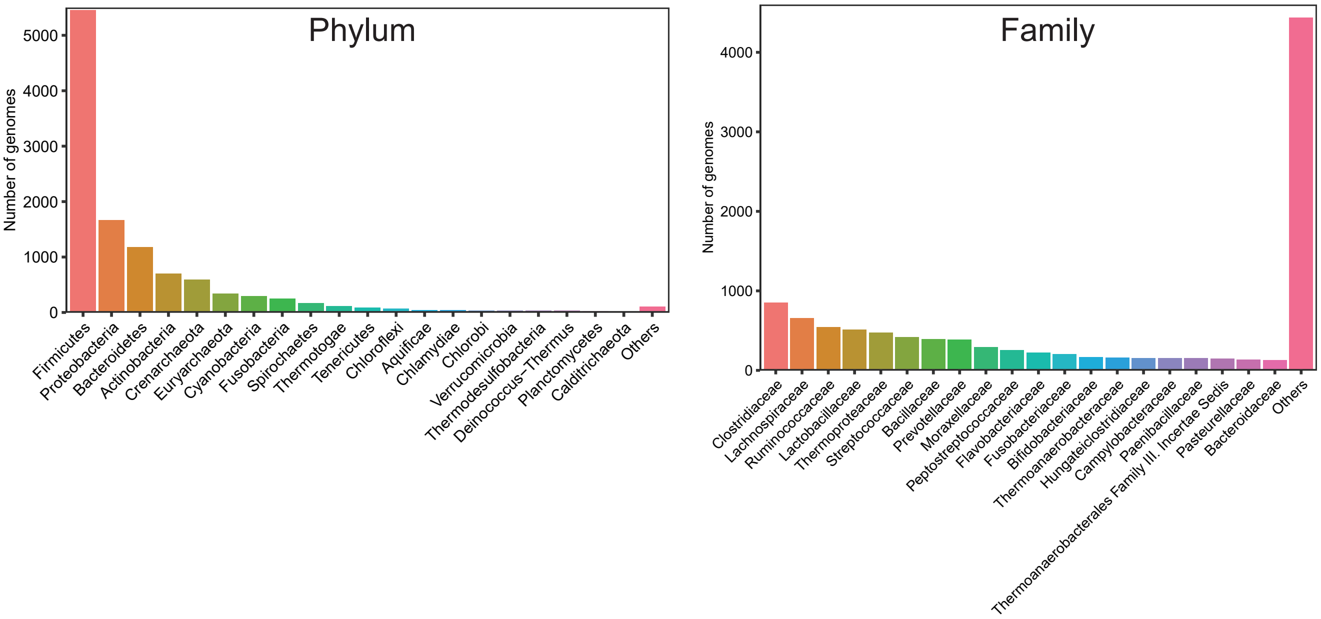

Figure S2 The host numbers of phages in IGPC at phylum and family levels.

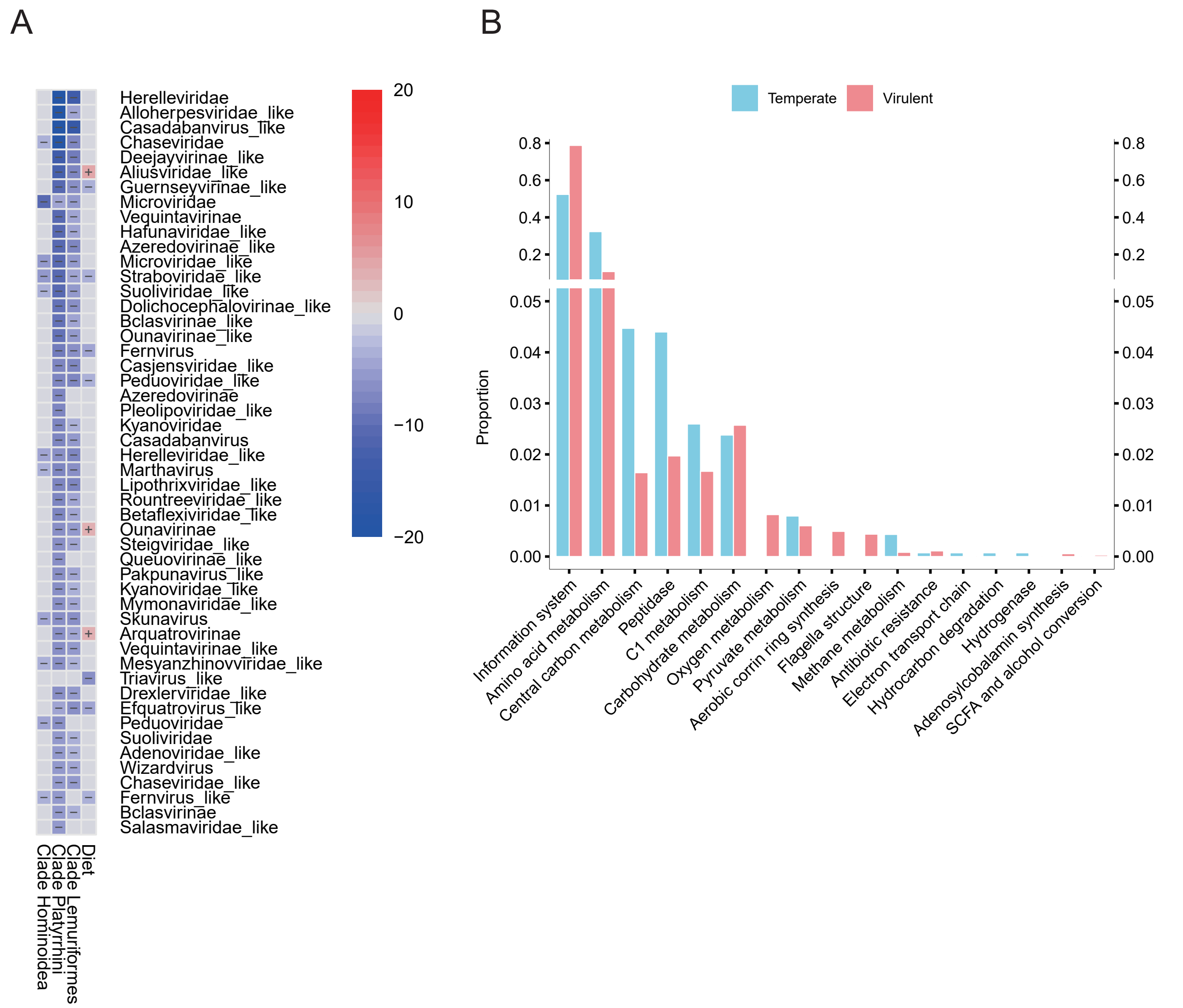

Figure S3 Gut phage diversity and AMG across wild NHP clades and dietary niches. (A)Top 50 phage families with significant associations in gut phages of NHPs with different clades and diet niche. Plus sign denotes significant enrichmentin nonfolivore for diet and non-Cercopithecidae for clade; minus sign indicates significant enrichment in folivore for diet and Cercopithecidae for clade. (B) The proportion of metabolism categories of AMGs in virulent and temperate phages.

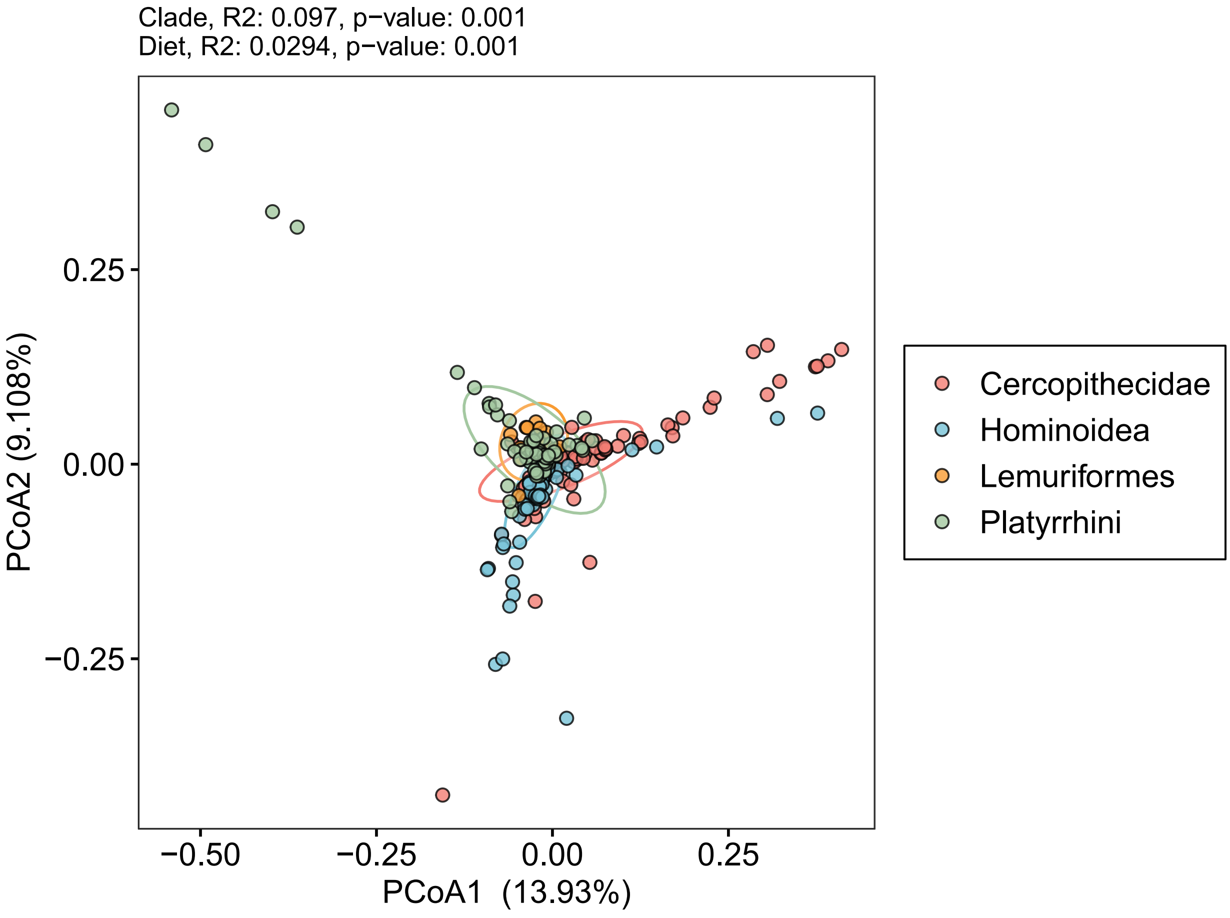

Figure S4. PCoA plot of weighted UniFrac distances of crAss-like phages across NHP samples. Colors represent host clades. PERMANOVA results: clade $R^2 = 0.0970$, $p = 0.001$; diet $R^2 = 0.0294$, $p = 0.001$.

Supplementary Methods

(a) Collection of NHP gut metagenomes

To present a comprehensive overview of the gut phageome in NHPs, we analyzed 454 gut metagenomes from 25 NHP species across 13 studies [1–13] (Fig. 1A and Table S1). Those samples that were explicitly described as "diseased" or "clinically abnormal" in these studies were excluded. These NHPs were grouped into captive (living in captivity, n = 135), wild (living independently in wild habitats, n = 286), and semiwild (living in wild habitats but receiving artificial food provisions, n = 33) populations (Table S2). According to their dietary habits, these NHPs were classified into folivores (11 NHP species) and nonfolivores (14 NHP species). We classified these NHPs into four clades according to genetic relationships: Hominoidea (apes, including 108 samples of 2 NHP species), Cercopithecidae (Old World monkeys, including 273 samples of 12 NHP species), Platyrrhini (New World monkeys, including 58 samples of 8 NHP species), and Lemuriformes (lemurs, including 15 samples of 3 NHP species).

(b) Assembly and identification of phage contigs

Adapters and low-quality reads in raw data were removed via Trimmomatic (v0.39) [14], a dependency of KneadData (v0.7.2) (https://github.com/biobakery/kneaddata). Host reads were removed via Bowtie2 (v2.5.4) [15], a dependency of KneadData, using the reference genomes of the hosts or their closely related species from NCBI genome datasets. High-quality metagenomic reads were assembled via MEGAHIT (v1.2.9) [16] with the option "--min-contig-len 1500" to obtain assembled contigs. The contigs were identified via VirSorter2 (v2.2.4) [17] with the option "--viral-gene-required --min-score 0.7 --min-length 1500" and the default "--include-groups dsDNAphage, ssDNA". These identified contigs were further verified via DeepVirFinder (v1.0) [18] with the option "-l 1500", and the contigs with score > 0.9 and p-value < 0.05 were regarded as putative phage contigs. The quality of the putative phage contigs was estimated via CheckV (v1.0.3) [19] using the "end to end" program. All contigs estimated as complete, high-quality, and medium-quality were clustered via CD-HIT (v4.6) [20] with the option "-c 0.95 -aS 0.85" to obtain a

nonredundant gut phage catalog, namely the IGPC.

(c) Annotations of phage lifestyles, prokaryote hosts, and taxa

Phage lifestyles (virulent and temperate) were predicted via PhaTYP [21] with the option "--len 1500". Phage hosts were predicted via CHERRY [22] with the option "--len 1500". Phage taxonomic labels at family and genus levels were predicted via PhaGCN2 (v2.3) [23] with the option "--len 1700" (the shortest length supported by this tool) and PhaGenus [24] with the option "--len 1500", respectively.

(d) AMG annotation of phage genomes

Phage AMGs were functionally annotated via DRAM-v (v1.5.0) [25] using KOfam and other default databases. The annotated result was filtered via the "distill" function in DRAM-v with the option "--remove_transposons --remove_fs".

(e) Comparison of the IGPC genomes with public viral databases

We compared the IGPC genomes with the NCBI viral reference genomes [26], the IMG/VR v4 database [27], and three human viral databases (Gut Virome Database (GVD) [28], Gut Phage Database (GPD) [29], and Metagenomic Gut Virus (MGV) database [30]). These sequences were locally aligned via BLASTN (v2.15.0+), and the average nucleotide identity (ANI) between pairwise sequences was calculated via the script "anicalc.py" of CheckV.

(f) Abundance quantification of phages and bacteria in metagenomes

The abundance of the IGPC phages in NHP gut metagenomes was quantified via CoverM (v0.7.0) [31] using the "coverm contig" mode and normalized using the transcripts per million (TPM) method. We downloaded 80 gut metagenomes of humans from industrialized (n = 39) or nonindustrialized (n = 41) countries [32–38]. The abundance of the IGPC phages in humans' gut metagenomes was quantified via CoverM.

The bacterial taxonomic label and abundance in NHP gut metagenomes were analyzed via MetaPhlAn4 (v4.1.1) [39] with the option "--ignore_eukaryotes --ignore_archaea" using the database "mpa_vOct22_CHOCOPhlAnSGB_202212" based on many unique microbial clade-specific marker genes.

(g) Identification and phylogenetic comparison of crAss-like phages from NHPs and humans

crAss-like phages, which were first detected in metagenomes, are prevalent in human gut and regarded as human fecal indicators [40]. To explore the distribution of crAss-like phages in NHP guts, we identified crAss-like phages in the IGPC following the method described in [40]. Protein-coding sequences of the IGPC phages were predicted with Prodigal (v2.6.3) [41] with the option "-p meta". Amino acid sequences of phages were aligned via BLASTP against the polymerase (UGP_018) and terminase (UGP_092) of the prototypical crAssphage (p-crAssphage, NC_024711.1) [42]. Phage genomes were considered to indicate crAss-like phages if they met the following criteria: (1) an E-value of less than 1E-05 against either the p-crAssphage polymerase or terminase; (2) an alignment length of at least 350 bp; and (3) a total genome length of greater than 70 kb. Using the predicted protein sequences, a rooted species tree was inferred by OrthoFinder (v2.5.5) [43] with the option "-S blast -M msa". Weighted UniFrac distances were calculated using the UniFrac function in the phyloseq package in R with the option "weighted = TRUE". Permutational multivariate analysis of variance (PERMANOVA) with 999 permutations was performed on the weighted UniFrac distance matrix using the adonis2 function in the vegan package in R, with host clade as the grouping factor.

We downloaded 922 crAss-like phage genomes from humans reported in previous studies [40,44]. To compare differences in crAss-like phages between humans and NHPs, multiple sequence alignments of their terminase protein sequences (UGP_092) were implemented via MAFFT (v7.526) [45] with the option "--auto". Based on the multiple sequence alignment, a phylogenetic tree was constructed via FastTree (v2.1.11) [46] and visualized via GraPhlAn (v1.1.3) [47]. Pairwise distances among crAss-like phages were calculated based on the terminase phylogenetic tree using the cophenetic.phylo function in the ape package in R. PERMANOVA with 999 permutations was then performed on the phylogenetic distance matrix, with host species as the grouping factor.

(h) Statistical analysis of data

Shannon indices of gut phages and bacteria in NHPs were calculated via the diversity function of the vegan package in R. The relationship between phage and bacterial Shannon diversity was examined using a linear mixed-effects model (LMM) with host species and data source study as random intercepts, and bacterial diversity as fixed effect. To assess the effects of NHP clade and dietary niche on gut phage diversity, composition, and function, we restricted these analyses to wild individuals only (n = 286). To compare differences in gut phage diversity and abundance among NHPs from different host clades and dietary niches, we used an LMM with host clade, dietary niche as fixed effects, and host species and data source study as random intercepts. Post-hoc pairwise comparisons were conducted using estimated marginal means (EMMs), with false discovery rate (FDR) correction. To assess the effects of NHP lifestyle, we used samples of each NHP species with different lifestyles from a single study, thus eliminating study-related batch effects. A linear model (LM) was used to assess the effect of lifestyle on phage diversity and abundance for each NHP species. For lifestyle factors with three levels, post-hoc pairwise comparisons were conducted using EMMs, with p-values adjusted by the FDR method; no adjustment was applied for two-level factors. The phage taxa and AMG categories with significant difference in abundance were analyzed via MaAsLin2 [48] with the option: max_significance = 0.05 and the defaults: normalization = "TSS", transform = "LOG", analysis_method = "LM", correction = "BH". For the analyses of host clade and dietary niche, we included host clade and dietary niche as fixed effects, and host species and data source study as random effects. For the analysis of lifestyle, we included lifestyle as the fixed effect. Principal coordinate analysis (PCoA) based on Bray-Curtis distance (using phage abundance) and Jaccard distance (using phage presence/absence) was performed using the vegan package in R. PERMANOVA with 999 permutations was performed using the adonis2 function of the vegan package in R to assess the significance of differences in gut phage community composition. Multivariate homogeneity of group dispersions was tested using the betadisper function in the vegan package in R to assess the significance of dispersion differences.

Reference

1. Amato KR *et al.* 2019 Evolutionary trends in host physiology outweigh dietary niche in structuring primate gut microbiomes. *ISME J.* **13**, 576–587. (doi:10.1038/s41396-018-0175-0)
2. Tung J *et al.* 2015 Social networks predict gut microbiome composition in wild baboons. *eLife* **4**, e05224. (doi:10.7554/eLife.05224)
3. Hicks AL *et al.* 2018 Gut microbiomes of wild great apes fluctuate seasonally in response to diet. *Nat. Commun.* **9**, 1786. (doi:10.1038/s41467-018-04204-w)
4. Li X *et al.* 2018 Establishment of a *Macaca fascicularis* gut microbiome gene catalog and comparison with the human, pig, and mouse gut microbiomes. *GigaScience* **7**, giy100. (doi:10.1093/gigascience/giy100)
5. Orkin JD, Campos FA, Myers MS, Cheves Hernandez SE, Guadamuz A, Melin AD. 2019 Seasonality of the gut microbiota of free-ranging white-faced capuchins in a tropical dry forest. *ISME J.* **13**, 183–196. (doi:10.1038/s41396-018-0256-0)
6. Campbell TP, Sun X, Patel VH, Sanz C, Morgan D, Dantas G. 2020 The microbiome and resistome of chimpanzees, gorillas, and humans across host lifestyle and geography. *ISME J.* **14**, 1584–1599. (doi:10.1038/s41396-020-0634-2)
7. Wang X, Wang Z, Pan H, Qi J, Li D, Zhang L, Shen Y, Xiang Z, Li M. 2021 Captivity influences the gut microbiome of *Rhinopithecus roxellana*. *Front. Microbiol.* **12**, 763022. (doi:10.3389/fmicb.2021.763022)
8. Rhoades NS, Cinco IR, Hendrickson SM, Slifka MK, Messaoudi I. 2022 Taxonomic and functional shifts in the perinatal gut microbiome of rhesus macaques. *Microbiol. Spectrum* **10**, e0081422. (doi:10.1128/spectrum.00814-22)
9. Xia W, Liu G, Wang D, Chen H, Zhu L, Li D. 2022 Functional convergence of Yunnan snub-nosed monkey and bamboo-eating panda gut microbiomes revealing the driving by dietary flexibility on mammal gut microbiome. *Comput. Struct. Biotechnol. J.* **20**, 685–699. (doi:10.1016/j.csbj.2022.01.011)
10. Yang S *et al.* 2022 The gut microbiome and antibiotic resistome of chronic

diarrhea rhesus macaques (*Macaca mulatta*) and its similarity to the human gut microbiome. *Microbiome* **10**, 29. (doi:10.1186/s40168-021-01218-3)

11. Li D, Xia W, Cui X, Zhao M, Huang K, Wang X, Shen J, Chen H, Zhu L. 2023 The putatively high-altitude adaptation of macaque monkeys: Evidence from the fecal metabolome and gut microbiome. *Evol. Appl.* **16**, 1708–1720. (doi:10.1111/eva.13595)
12. Sun Y, Yu Y, Wu A, Zhang C, Liu X, Qian C, Li J, Ran J. 2023 The composition and function of the gut microbiota of Francois' langurs (*Trachypithecus francoisi*) depend on the environment and diet. *Front. Microbiol.* **14**, 1269492. (doi:10.3389/fmicb.2023.1269492)
13. Zhang D *et al.* 2025 Multi-omic profiling reveals distinct gut microbial and metabolic landscapes in golden snub-nosed monkeys under contrasting conservation strategies. *npj Biofilms Microbiomes* **11**, 209. (doi:10.1038/s41522-025-00836-1)
14. Bolger AM, Lohse M, Usadel B. 2014 Trimmomatic: a flexible trimmer for illumina sequence data. *Bioinformatics* **30**, 2114–2120. (doi:10.1093/bioinformatics/btu170)
15. Langmead B, Salzberg SL. 2012 Fast gapped-read alignment with bowtie 2. *Nat. Methods* **9**, 357–359. (doi:10.1038/nmeth.1923)
16. Li D, Liu C-M, Luo R, Sadakane K, Lam T-W. 2015 MEGAHIT: an ultra-fast single-node solution for large and complex metagenomics assembly via succinct de bruijn graph. *Bioinformatics* **31**, 1674–1676. (doi:10.1093/bioinformatics/btv033)
17. Guo J *et al.* 2021 VirSorter2: a multi-classifier, expert-guided approach to detect diverse DNA and RNA viruses. *Microbiome* **9**, 37. (doi:10.1186/s40168-020-00990-y)
18. Ren J, Song K, Deng C, Ahlgren NA, Fuhrman JA, Li Y, Xie X, Poplin R, Sun F. 2020 Identifying viruses from metagenomic data using deep learning. *Quant. Biol.* **8**, 64–77. (doi:10.1007/s40484-019-0187-4)
19. Nayfach S, Camargo AP, Schulz F, Eloe-Fadrosh E, Roux S, Kyrpides NC. 2021

CheckV assesses the quality and completeness of metagenome-assembled viral genomes. *Nat. Biotechnol.* **39**, 578–585. (doi:10.1038/s41587-020-00774-7)

20. Fu L, Niu B, Zhu Z, Wu S, Li W. 2012 CD-HIT: accelerated for clustering the next-generation sequencing data. *Bioinformatics* **28**, 3150–3152. (doi:10.1093/bioinformatics/bts565)
21. Shang J, Tang X, Sun Y. 2023 PhaTYP: predicting the lifestyle for bacteriophages using BERT. *Briefings Bioinf.* **24**, bbac487. (doi:10.1093/bib/bbac487)
22. Shang J, Sun Y. 2022 CHERRY: a Computational metHod for accuratE pRediction of virus-pRokarYotic interactions using a graph encoder-decoder model. *Briefings Bioinf.* **23**, bbac182. (doi:10.1093/bib/bbac182)
23. Jiang J-Z *et al.* 2023 Virus classification for viral genomic fragments using PhaGCN2. *Briefings Bioinf.* **24**, bbac505. (doi:10.1093/bib/bbac505)
24. Guan J, Peng C, Shang J, Tang X, Sun Y. 2023 PhaGenus: genus-level classification of bacteriophages using a Transformer model. *Briefings Bioinf.* **24**, bbad408. (doi:10.1093/bib/bbad408)
25. Shaffer M *et al.* 2020 DRAM for distilling microbial metabolism to automate the curation of microbiome function. *Nucleic Acids Res.* **48**, 8883–8900. (doi:10.1093/nar/gkaa621)
26. Brister JR, Ako-Adjei D, Bao Y, Blinkova O. 2015 NCBI viral genomes resource. *Nucleic Acids Res.* **43**, D571-577. (doi:10.1093/nar/gku1207)
27. Camargo AP *et al.* 2023 IMG/VR v4: an expanded database of uncultivated virus genomes within a framework of extensive functional, taxonomic, and ecological metadata. *Nucleic Acids Res.* **51**, D733–D743. (doi:10.1093/nar/gkac1037)
28. Gregory AC, Zablocki O, Zayed AA, Howell A, Bolduc B, Sullivan MB. 2020 The gut virome database reveals age-dependent patterns of virome diversity in the human gut. *Cell Host Microbe* **28**, 724-740.e8. (doi:10.1016/j.chom.2020.08.003)
29. Camarillo-Guerrero LF, Almeida A, Rangel-Pineros G, Finn RD, Lawley TD. 2021 Massive expansion of human gut bacteriophage diversity. *Cell* **184**, 1098-1109.e9. (doi:10.1016/j.cell.2021.01.029)

30. Nayfach S *et al.* 2021 Metagenomic compendium of 189,680 DNA viruses from the human gut microbiome. *Nat. Microbiol.* **6**, 960–970. (doi:10.1038/s41564-021-00928-6)
31. Aroney STN, Newell RJP, Nissen JN, Camargo AP, Tyson GW, Woodcroft BJ. 2025 CoverM: read alignment statistics for metagenomics. *Bioinformatics* **41**, btaf147. (doi:10.1093/bioinformatics/btaf147)
32. Rampelli S *et al.* 2015 Metagenome sequencing of the Hadza hunter-gatherer gut microbiota. *Curr. Biol.* **25**, 1682–1693. (doi:10.1016/j.cub.2015.04.055)
33. Vogtmann E *et al.* 2016 Colorectal cancer and the human gut microbiome: reproducibility with whole-genome shotgun sequencing. *PLOS One* **11**, e0155362. (doi:10.1371/journal.pone.0155362)
34. Obregon-Tito AJ *et al.* 2015 Subsistence strategies in traditional societies distinguish gut microbiomes. *Nat. Commun.* **6**, 6505. (doi:10.1038/ncomms7505)
35. Wen C *et al.* 2017 Quantitative metagenomics reveals unique gut microbiome biomarkers in ankylosing spondylitis. *Genome Biol.* **18**, 142. (doi:10.1186/s13059-017-1271-6)
36. Costea PI *et al.* 2017 Subspecies in the global human gut microbiome. *Mol. Syst. Biol.* **13**, 960. (doi:10.15252/msb.20177589)
37. Asnicar F *et al.* 2017 Studying vertical microbiome transmission from mothers to infants by strain-level metagenomic profiling. *mSystems* **2**, e00164-16. (doi:10.1128/mSystems.00164-16)
38. Liu W *et al.* 2016 Unique features of ethnic Mongolian gut microbiome revealed by metagenomic analysis. *Sci. Rep.* **6**, 34826. (doi:10.1038/srep34826)
39. Blanco-Míguez A *et al.* 2023 Extending and improving metagenomic taxonomic profiling with uncharacterized species using MetaPhlAn 4. *Nat. Biotechnol.* **41**, 1633–1644. (doi:10.1038/s41587-023-01688-w)
40. Guerin E *et al.* 2018 Biology and taxonomy of crAss-like bacteriophages, the most abundant virus in the human gut. *Cell Host Microbe* **24**, 653-664.e6. (doi:10.1016/j.chom.2018.10.002)
41. Hyatt D, Chen G-L, Locascio PF, Land ML, Larimer FW, Hauser LJ. 2010

Prodigal: prokaryotic gene recognition and translation initiation site identification. *BMC Bioinf.* **11**, 119. (doi:10.1186/1471-2105-11-119)

42. Dutilh BE *et al.* 2014 A highly abundant bacteriophage discovered in the unknown sequences of human faecal metagenomes. *Nat. Commun.* **5**, 4498. (doi:10.1038/ncomms5498)
43. Emms DM, Kelly S. 2019 OrthoFinder: phylogenetic orthology inference for comparative genomics. *Genome Biol.* **20**, 238. (doi:10.1186/s13059-019-1832-y)
44. Yutin N, Benler S, Shmakov SA, Wolf YI, Tolstoy I, Rayko M, Antipov D, Pevzner PA, Koonin EV. 2021 Analysis of metagenome-assembled viral genomes from the human gut reveals diverse putative CrAss-like phages with unique genomic features. *Nat. Commun.* **12**, 1044. (doi:10.1038/s41467-021-21350-w)
45. Katoh K, Standley DM. 2013 MAFFT multiple sequence alignment software version 7: improvements in performance and usability. *Mol. Biol. Evol.* **30**, 772–780. (doi:10.1093/molbev/mst010)
46. Price MN, Dehal PS, Arkin AP. 2010 FastTree 2--approximately maximum-likelihood trees for large alignments. *PLOS One* **5**, e9490. (doi:10.1371/journal.pone.0009490)
47. Asnicar F, Weingart G, Tickle TL, Huttenhower C, Segata N. 2015 Compact graphical representation of phylogenetic data and metadata with GraPhlAn. *PeerJ* **3**, e1029. (doi:10.7717/peerj.1029)
48. Mallick H *et al.* 2021 Multivariable association discovery in population-scale meta-omics studies. *PLoS Comput. Biol.* **17**, e1009442. (doi:10.1371/journal.pcbi.1009442)
